# Deep eutectic solvent enhances antibacterial activity of a modular lytic enzyme against *Acinetobacter baumannii*

**DOI:** 10.1038/s41598-024-80440-z

**Published:** 2025-01-15

**Authors:** Aleksandra Maria Kocot, Tomasz Swebocki, Karolina Ciemińska, Adrianna Łupkowska, Małgorzata Kapusta, Dennis Grimon, Ewa Laskowska, Anna-Karina Kaczorowska, Tadeusz Kaczorowski, Rabah Boukherroub, Yves Briers, Magdalena Plotka

**Affiliations:** 1 Laboratory of Extremophiles Biology, Department of Microbiology, Faculty of Biology, University of Gdansk, Wita Stwosza 59, Gdansk, 80-308 Poland; 2 Univ. Lille, CNRS, Univ. Polytechnique Hauts-de-France, UMR 8520 IEMN – Institut d’Electronique de Microélectronique et de Nanotechnologie, Lille, 59000 France; 3 Department of General and Medical Biochemistry, Faculty of Biology, University of Gdansk, Wita Stwosza 59, Gdansk, 80-308 Poland; 4 Bioimaging Laboratory, Faculty of Biology, University of Gdansk, Wita Stwosza 59, Gdansk, 80-308 Poland; 5 Department of Biotechnology, Faculty of Bioscience Engineering, Ghent University, Valentin Vaerwyckweg 1, Ghent, 9000 Belgium; 6 Collection of Plasmids and Microorganisms | KPD, Faculty of Biology, University of Gdansk, Wita Stwosza 59, Gdansk, 80-308 Poland; 7 Institute of Nanotechnology and Materials Engineering, Faculty of Applied Physics and Mathematics, Gdańsk University of Technology, Narutowicza 11/12, Gdansk, 80-233 Poland

**Keywords:** Modular lytic enzyme, Endolysin, Deep eutectic solvent, Synergism, Antibacterial effect, Biofilm, Persisters, Antimicrobials, Antimicrobial resistance, Microbiology, Proteins

## Abstract

In this study, we evaluated the combined effect between MLE-15, a modular lytic enzyme composed of four building blocks, and reline, a natural deep eutectic solvent. The bioinformatic analysis allowed us to determine the spatial architecture of MLE-15, whose components were bactericidal peptide cecropin A connected via a flexible linker to the cell wall binding domain (CBD) of mesophilic 201ϕ2 − 1 endolysin and catalytic domain (EAD) of highly thermostable Ph2119 endolysin. The modular enzyme showed high thermostability with the melting temperature of 93.97 ± 0.38 °C, significantly higher than their natural counterparts derived from mesophilic sources. The minimum inhibitory concentration (MIC) of MLE-15 was 100 µg/mL for a panel of Gram-positive and Gram-negative bacteria, while the MIC of reline ranged from 6.25 to 25% v/v for the same strains. The addition of reline effectively reduced the MIC of MLE-15 from 100 µg/mL to 3.15–50 µg/mL. This combination displayed additive effects for most strains and synergism for extensively antibiotic-resistant *Acinetobacter baumannii* and *Bacillus subtilis*. The subsequent evaluation revealed that MLE-15 eliminated planktonic cells of *A. baumannii* RUH134, but was ineffective against matured biofilms. However, combined with reline, MLE-15 reduced the bacterial load in the matured biofilm by 1.39 log units. Confocal fluorescence microscopy indicated that reline damaged the structure of the biofilm, allowing MLE-15 to penetrate it. Additionally, MLE-15 and its combination with reline eradicated meropenem-persistent cells of *A. baumannii* RUH134. Effectiveness in lowering the MIC value of MLE-15 as well as protection against antibiotic-tolerant persister cells, indicate that MLE-15 and reline combination is a promising candidate for effective therapies in bacterial infections, which is especially important in the light of the global crisis of antimicrobial resistance.

**Supplementary Information:**

The online version contains supplementary material available at 10.1038/s41598-024-80440-z.

## Introduction

Amid the global crisis of antimicrobial resistance (AMR) driven by the increase in multidrug-resistant bacteria, the scientific community’s primary objective is to discover new and effective antibacterial agents^1,2^. Joint studies by the Institute for Health Metrics and Evaluation (IHME) and the University of Oxford revealed that in 2019 alone, approximately 13.66 million people died from microbe-induced sepsis. Over 65% of these deaths (8.88 million) were attributed to bacterial diseases, with 4.95 million associated with antimicrobial resistance^1^. The group of pathogens characterized by the highest resistance to currently available therapeutics is called ESKAPEE, an acronym for *Enterococcus faecium*, *Staphylococcus aureus*, *Klebsiella pneumoniae*, *Acinetobacter baumannii*, *Pseudomonas aeruginosa*, *Enterobacter* spp., and *Escherichia coli*^3,4^.

A particularly challenging aspect of AMR is the formation of bacterial biofilm, a complex bacterial population that exhibits significantly greater resistance to antibacterial agents, including antibiotics, than planktonic bacteria^5–7^. While most antibacterial agents show the highest activity against cells in the outer layer of biofilms, the presence of extracellular polymeric substances (EPS) significantly reduces their efficacy in the inner layers^7,8^. The frequent presence of persister cells, a subpopulation that become dormant in the presence of antibacterial agents and regain physiological activity once exposure ceases^9,10^, in biofilms poses an additional challenge. This can lead to the rebuilding of biofilms, potentially causing recurrent infections^11,12^. Therefore, in pursuit of effective strategies counteracting AMR, it is imperative to explore novel approaches and assess their efficacy against planktonic cells and biofilms while expanding the study to include persister cells.

**Fig. 1:**
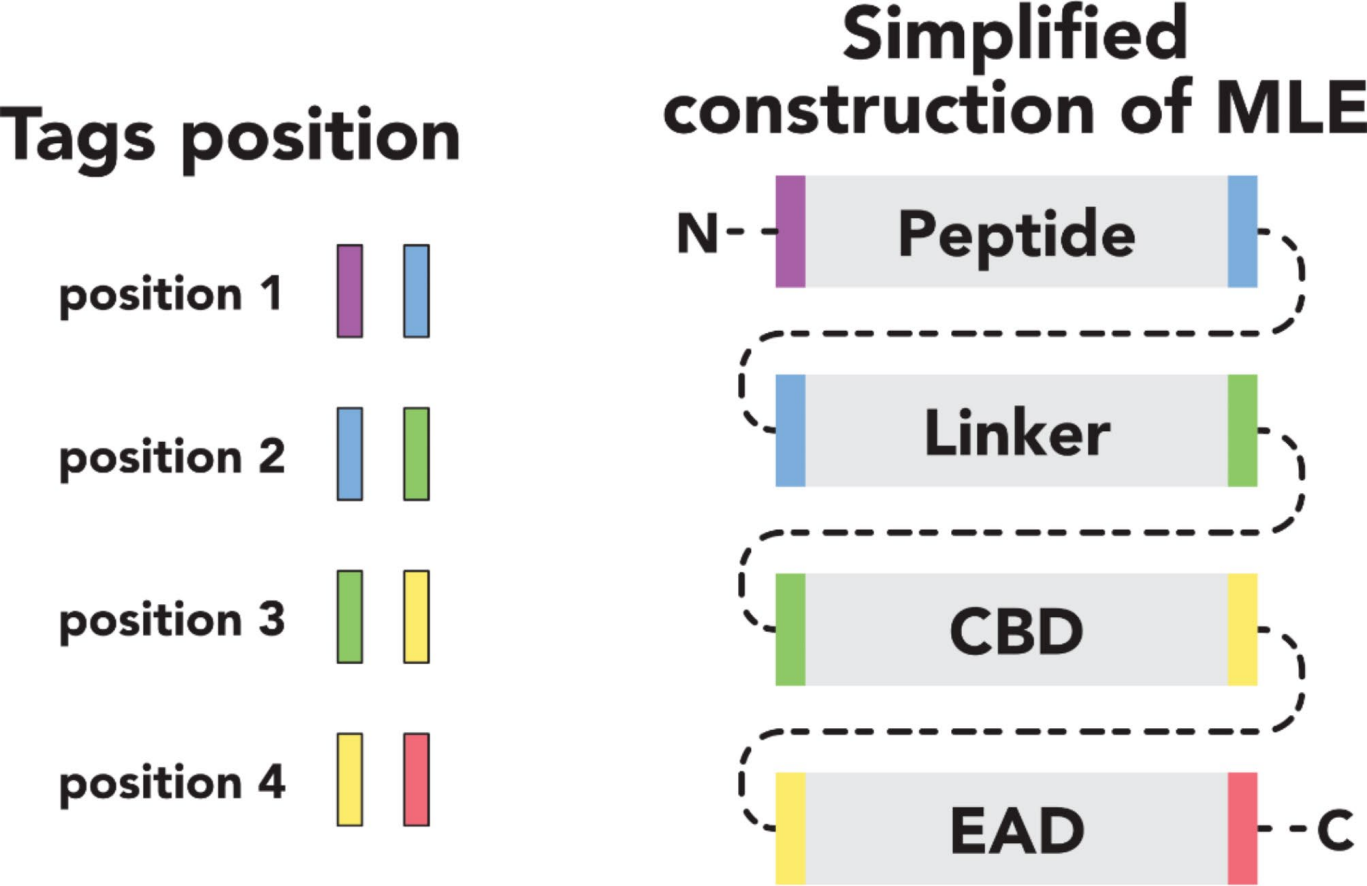
The MLEs construction scheme includes CBD – cell-wall binding domain and EAD – enzymatically active domain (according to Gerstmans et al., 2020^18^).

Recently, an alternative approach in antibacterial therapies focused on the application of enzybiotics (enzyme-based antibacterials) has emerged^13,14^. Among these enzymes, phage-originating endolysins, responsible for the degradation of the peptidoglycan layer of the bacterial cell wall, draw particular attention^15,16^. Most endolysins are effective against Gram-positive bacteria but have limited efficacy against Gram-negative bacteria^17,18^. Generally, endolysins active against Gram-negative bacteria have a globular structure with a single enzymatically active (catalytic) domain (EAD). In contrast, endolysins targeting Gram-positive bacteria usually have a multidomain, modular architecture^18^. The structure of modular endolysins or modular lytic enzymes (MLE) is determined by the presence of at least one EAD and cell wall-binding domain (CBD) connected by a linker^18^. The limited activity of endolysins against Gram-negative bacteria is a direct result of the presence of an outer membrane^18^. The outer membrane acts as a chemo-physical barrier that can be penetrated by molecules smaller than 600 Da, which enter through embedded porins. However, it presents an obstacle to larger molecules, such as endolysins^17^. A significant portion of the ESKAPEE group is represented by Gram-negative bacteria. Therefore, it is necessary to search for solutions to improve the efficacy of lysins against Gram-negative strains^18^. One promising solution is protein engineering aimed at obtaining modular lytic enzymes (MLEs) with desirable properties. Due to their modular structure (Fig. 1), endolysins of phage origin are well-suited for domain shuffling.

Moreover, the efficacy of endolysins against Gram-negative bacteria can be augmented by fusing them with an antimicrobial peptide (AMP). AMPs are typically small (10–60 amino acids), predominantly cationic peptides found abundantly in nature^19^. They are often considered a promising substitute for traditional antibiotics^20,21^. Among AMPs, a category referred to as outer membrane permeabilizing peptides (OMPs) exists^22^. Fusing an OMP with endolysin produces an MLE with potential activity against Gram-negative bacteria.

In 2020, the high-throughput VersaTile method for modular enzyme engineering was described^18^. This innovative DNA assembly method takes the advantage of the modular structure of endolysins and relies on their fusion with OMP. Using VersaTile, it is possible to replace and shuffle an infinite number of EADs, CBDs, linkers, and many OMPs^18^ allowing for quick generation of engineered lysin libraries and rapid screening to identify variants with high antibacterial activity^18^. In the pioneering publication on VersaTile^18^, a lead variant 1D10 consisting of OMP7–linker1–CBD6–EAD20 completely inhibited the growth of *A. baumannii* NCTC13423 and RUH134 in simulated human physiological conditions (50% human serum). Moreover, it slowed biofilm formation in a porcine skin wound infection model^18^. Obtaining MLE with high antibacterial activity against *A. baumannii* prompted us to use the VersaTile method to obtain variants containing EAD from the thermostable endolysin Ph2119 discovered in our laboratory^23^.

Another example of innovative compounds that has not yet been used in combination with endolysins, are natural deep eutectic solvents (NADESs)^24^. NADESs are an example of green chemistry materials, and in addition to water and lipids, constitute the third class of solvents, providing an alternative to conventional volatile and organic solvents^25,26^. These viscous liquids are formed as a result of the formation of an intricate hydrogen bond network between hydrogen bond donors (such as amines, sugars, carboxylic acids, and alcohols) and hydrogen bond acceptors (e.g., quaternary ammonium salts choline chloride or betaine)^25,27^. NADESs are formulated by grinding, sonication, or heating of parent substances and can be synthesized without using solvents or catalysts. They do not require any further purification^27^. To date, DESs have been used mainly in chemical transformations as substitutes for chemical solvents or in (bio)medicine as drug delivery systems or enhancers of antibiotic activity^28–30^. Depending on the selection of parent substances, NADESs may exhibit antibacterial properties, as shown in previous studies^31,32^. NADESs have been implemented to enhance the activity of protein-based drugs, such as tumor necrosis factor (TNF⍺) antibody^33^, glucagon-like peptide 1, (GLP-1)^34^ and insulin^35^. However, to our knowledge, there is currently no information on the synergy between NADESs and antimicrobial agents other than conventional small-molecule antibiotics. Moreover, the mechanism of action of NADES against biofilms and the effectiveness against persister cells have not yet been established.

Therefore, in response to the urgent need for new and effective alternatives to conventional antibiotics, it seems imperative to evaluate the antibacterial properties of MLE. Considering the above, the aim of this study was to investigate the antibacterial effect of modular lytic enzyme (MLE-15) containing the EAD of Ph2119 thermostable endolysin of *Thermus scotoductus* phage 2119, and reline as a representative of deep eutectic solvents alone and in combination against planktonic bacteria, biofilms and persister cells.

## Materials and methods

### Bacterial strains, media and culture conditions

All Gram-negative (*Pseudomonas aeruginosa* PAO1, *Pseudomonas fluorescens* DSM 50090, *Klebsiella pneumoniae* P3, *Escherichia coli* P4-7250, *Acinetobacter baumannii* CRAB, and *Acinetobacter baumannii* RUH134) and Gram-positive strains (*Staphylococcus aureus* Z8, *Enterococcus faecalis* Z9, and *Bacillus subtilis* subsp. *spizizenii* ATCC 6633) used in the study were supplied either by Leibniz-Institut DSMZ – Deutsche Sammlung von Mikroorganismen und Zellkulturen GmbH, Germany, the American Type Culture Collection (ATCC), or by the collection of bacterial cultures of the Department of Microbiology, University of Gdansk, Poland. The strains were stored in the form of glycerol stocks at − 80℃. For each analysis, an overnight culture was prepared in fresh Luria-Bertani (LB) broth or Mueller Hinton (MH) broth (Merck, Germany) and incubated at 37℃.

### Preparation of reline

Reline was prepared at a molar ratio of 1:2 choline chloride (Sigma Aldrich, C1879-500G) and urea (Sigma Aldrich, U5378-1KG)^36^. DES was obtained by mixing and heating parent substances slowly, with occasional vortex stirring, until the content of the vessel was completely liquefied. After the substances melted, heating was stopped, and the obtained DES was let to cool down naturally to room temperature. The prepared DES was stored dry at room temperature until the experiments were performed.

### Protein structure prediction

**Fig. 2:**
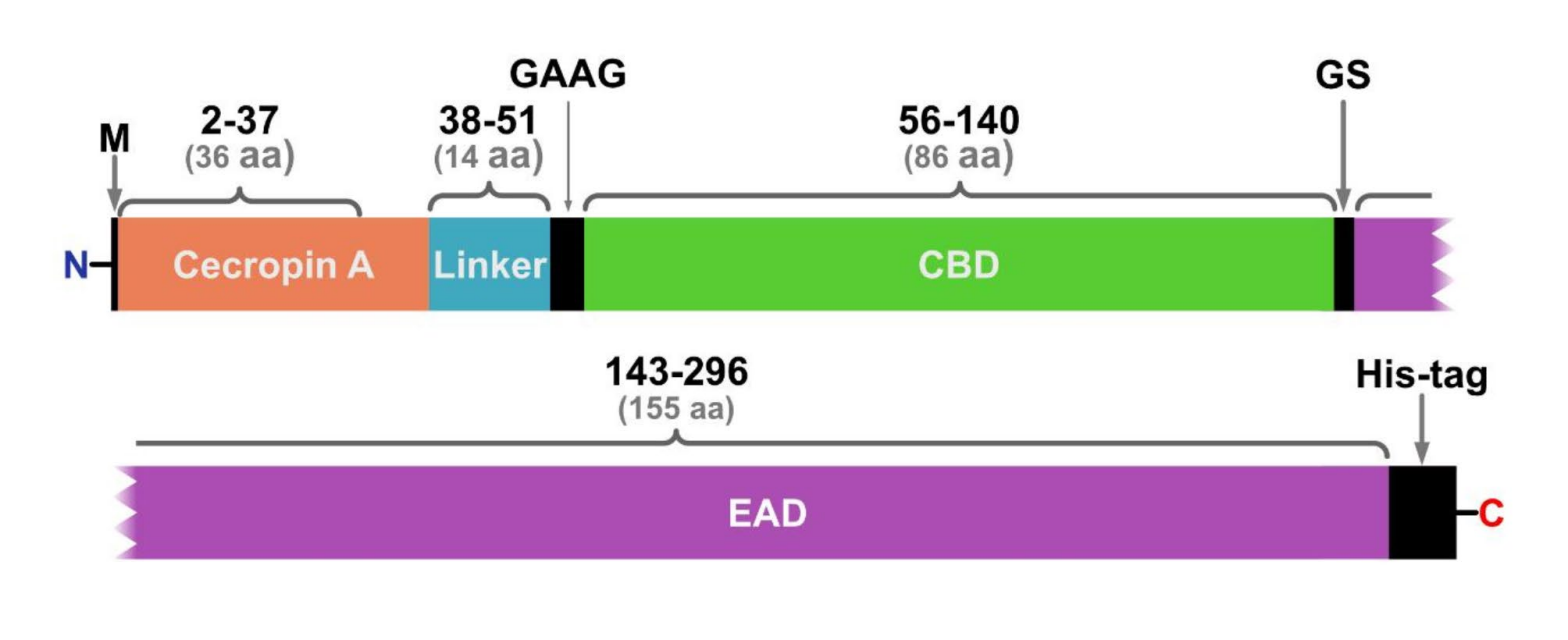
Block model of MLE-15 (M, start codon; GAAG/GS, cleavage sites).

VersaTile method used to obtain the library of MLEs was in details described by Gerstmans et al.^18^. In preliminary screening by bacteria growth inhibitory assay^18^, out of 200 tested variants, MLE-15 completely inhibited bacterial growth of *A. baumannii* RUH134 and *P. aeruginosa* PAO1 (data not shown) and was assigned as a lead variant. This variant consisted of cecropin A (OMP), linker 2, the CBD of endolysin 201phi2-1, and the EAD Ph2119 (thermostable endolysin module) (Table 1; Fig. 2).

**Table 1:** Components of the modular lytic enzyme – MLE-15.

Code	Position	Sequential position	Description	Origin	Accession number	Ref.
OMP7	1	Gly2 - Lys37	Cecropin A	*Aedes aegypti (yellow fever mosquito)*	AAF59831	[37]
Link2	2	Gly38-Ala51	Flexible median linker	*In silico* design	-	[18]
CBD5	3	Ile56- Gln140	201phi2-1gp229-CBD, CBD of endolysin 201phi2-1	*Pseudomonas* phage *201phi2-1*	YP_001956952	[38]
EAD29	4	Arg143- Lys296	Ph2119	*Thermus* phage 2119	HF20915.1	[23]
**The amino acid sequence of MLE-15 (304 aa)**						
MGGLKKLGKKLEGAGKRVFNAAEKALPVVAGAKALRKGAGAGAGAGAGAGAGAAGILKNGSKGDDVIRLQRKLIGLGYSVKDDGVFGDNTEKAVKAVQLRFNLKDDGIVGNNTWAVLDTPTTTRPALTDKDYQWAADYLQGSRILEPWNRWYRQKGVYRIRGTPPHYIVLHHTAGPVDQAPEVIRDFHEKGRGWPHIGYHYLVYQDGRVYKTLPNNAIPICVREFNPVSLCIAAVGDFSQGPAWPDNAPGWKALLELKDALVKAYPKAVLVLHKELTQTTCPGVLSWGMVAEKGGKKYHHHHHH						

Using the amino acid sequences of the individual fragments, we aimed to construct a 3D model of MLE-15 with AlphaFold v2.3.2^39^, ESMFold_advanced^40^, DeepFold^41^, Phyre2^42^, OmegaFold^43^ and MODELLER^44^. Predictions were carried out on the Google Colab platform for most tools, with MODELLER executed locally and DeepFold on the Zhang Group platform. Structural homologs of MLE-15 used in MODELLER were found with Foldseek^45^. Side chain optimization was performed with GalaxyRefine^46,47^. Modeling accuracy was evaluated in Chimera 1.16^48^ using superposition to the existing model of the Ph2119 domain (PDB entry: 6SU5). To evaluate the cecropin A fragment, a structural model was predicted with ESMFold and OmegaFold. Foldseek was implemented for searching for similar peptides and calculating TM-scores. Protein visualization was also performed in Chimera 1.16.

### Overproduction and purification of modular lytic enzyme

MLE-15 was overexpressed in the *E. coli* BL21(DE3) pRIL strain. For this purpose, 1 L of LB supplemented with kanamycin (100 µg/mL) and chloramphenicol (30 µg/mL) was used. An overnight culture of *E. coli* harboring the recombinant plasmid (pVTD-MLE-15) was diluted 100-fold and grown until OD_600_ of 0.4 − 0.5. Then, the protein overproduction was induced with 1 mM isopropyl-β-d-thiogalactopyranoside (IPTG) following the incubation for 4 h at 37 °C. Finally, bacterial cells were harvested by centrifugation (6,000 × g, 20 min, 4 °C), suspended in NPI-10 buffer (50 mM NaH_2_PO_4_, pH 8.0, 300 mM NaCl, 10 mM imidazole, 0.1% Triton X-100, and 10% (v/v) glycerol) and stored at − 80 °C.

Subsequently, purification using the ÄKTA Pure Protein Purification System (Cytiva) was performed. First, cells were disrupted by sonication (30 bursts of 10 s at an amplitude of 12 μm, 4℃). Then, the cell lysates were centrifuged (11 000 × g, 20 min, 4 °C) and heated (20 min, 65℃). After that, the supernatant with His-tagged MLE-15 was subjected to cobalt-based immobilized metal affinity chromatography with 1 mL HiTrap TALON crude columns (Cytiva), washed (NPI buffer with 20 mM imidazole), and eluted (NPI buffer without 0.1% Triton X-100 with 150 mM imidazole). Fractions with pure protein were dialyzed overnight against 20 mM HEPES buffer (pH 7.4) supplemented with 50% glycerol at 4℃. Finally, protein homogeneity was evaluated by sodium dodecyl sulfate-polyacrylamide gel electrophoresis (SDS-PAGE), and the protein concentration was determined via Bradford assay using bovine serum albumin as a standard.

### Nano differential scanning fluorimetry

MLE-15 sample at a protein concentration of 0.8 mg/mL (in 20 mM HEPES, pH 7.4, 10% glycerol, 25 mM NaCl buffer) was equilibrated to room temperature and centrifuged (10,000 × g, 1 min) to remove any residual air bubbles. Subsequently, the sample was loaded in four replicates into Prometheus standard capillaries and sealed with capillary sealing paste (NanoTemper Technologies) to prevent evaporation. The measurements were conducted at a temperature range from 20 to 110 °C with a 1 °C interval using the Prometheus NT.48 AGOHTU (NanoTemper Technologies) to determine the protein’s thermal stability. During the melting scans, the intrinsic protein fluorescence at 330 nm (F350) and 350 nm (F350) signal was recorded and further using the PR.ThermControl and PR.Stability Analysis software, respectively.

### Determination of minimum inhibitory concentration (MIC)

MIC was determined according to Gerstmans et al.^18^ with modifications^49^. Briefly, an overnight culture of bacteria was diluted 100-fold in MH broth and grown to an OD_600_ = 0.5. After that, the cultures were diluted 5 000-fold in MH broth. Four hundred microliters of MLE-15 (200 µg/mL) or reline (50% v/v) prepared in MH broth were transferred onto first row of 96-well microtiter plates. To further wells two hundred microliters of MH was added and 2-fold dilutions of MLE-15 or reline were prepared. For experiments with MLE-15 and reline, reline was mixed with MH broth to achieve the sub-MIC concentration of 4.5% v/v. Solution was transferred onto 96-well microtiter plate. MLE-15 was added to the first row to final concentration of 200 µg/mL and 2-fold serial dilutions of MLE-15 were prepared as described above. Then, 20 µL of adjusted bacteria were added to each well (final count of approx. 2 × 10^5^ cells). Sterile MH broth (200 µL) and medium inoculated with bacteria were used as negative (sterility) and positive (growth) controls, respectively. The samples were then incubated for 18 h at 37 °C, and the MIC values were determined as the lowest concentration that completely inhibited growth visible to the naked eye.

### Antibacterial test

Antibacterial tests were performed followed the protocol of Szadkowska et al., 2023^50^. All bacterial strains were grown in LB broth at 37 °C until the OD_600_ = 0.5. One milliliter of each culture was centrifuged (4,000 × g, 15 min, 20 °C) and washed with 20 mM HEPES buffer (pH 7.4) or saline (0.85% NaCl) for MLE-15 and reline, respectively. Subsequently, the harvested cells (10-fold dilution, approx. 10^7^ cells) were resuspended in either 20 mM HEPES (pH 7.4) or saline supplemented with MLE-15 or reline (MIC value applied based on the results in point 2.6). 20 mM HEPES buffer (pH 7.4) was used as a negative control. The samples were incubated for 1.5 h at 37 °C. Then, the samples were centrifuged (4,000 × g, 15 min, 20 °C), washed with saline, suspended, serially diluted, plated onto Luria-Bertani agar (LA) plates, and incubated for 24 h at 37 °C. After incubation, the colonies were counted, and the results were expressed as log CFU/mL. The antibacterial tests with use of buffers with different tonicity (20 mM HEPES buffer pH 7.4 without or supplemented with 30 mM NaCl (hypotonic buffer), 150 mM NaCl (isotonic buffer), and 350 mM NaCl (hypertonic buffer) were performed according to Vázquez et al., 2024^51^, but because of 600 µL reaction volume, tests were performed in Eppendorf tubes not in sterile 96-deep-well plates.

### Biofilm eradication assay

An overnight culture of bacteria was refreshed in LB broth and adjusted to OD_600_ = 0.5. A 500 µL of adjusted culture was then transferred to a 24-well microtiter plate and 500 µL of LB broth was added and mixed with the culture. The inoculated plate was incubated at 37 °C for 24 h. The medium was removed, and the biofilm was gently washed twice with 1 mL of 20 mM HEPES buffer (pH 7.4) or saline for MLE-15 and reline, respectively. Next, biofilms were treated with 500 µL of MLE-15 or reline at the concentration of 2×MIC. The solutions of MLE-15 and reline were prepared in HEPES or saline respectively. Five hundred µL of HEPES buffer was used as a negative control. Samples were incubated at 37 °C for 3 h. Then, the supernatant was removed, and the biofilm was gently washed twice with saline. Next, the biofilm was harvested with a sterile swab and placed in 1 mL of saline. The samples were serially diluted, and appropriate dilutions were plated onto LB agar plates and incubated at 37 °C for 24 h. After incubation, the colonies were counted, and the results were expressed as log CFU/mL^5^.

### Confocal laser scanning microscopy (CLSM)

Biofilms of *A. baumannii* RUH134 were prepared in the LabTek™ chamber slide system according to Kocot and Olszewska^52^. Initially, biofilms were prepared as described in Sect. 2.8. After treatment (MLE-15: 2×MIC, reline: 2×MIC, combination: MLE-15 1×MIC and reline sub-MIC) and subsequent washing, they were stained using LIVE/DEAD Bac Light Bacterial Viability Kit (Thermo Fisher Scientific; L7012) using 1.5 µL of SYTO 9 and 1.5 µL of propidium iodide (PI) diluted in 1 mL of saline. Biofilms were incubated at room temperature for 20 min without light. Next, the staining mixture was removed, and the chambers were detached from the slide. Then, a coverslip was placed on the gasket and mounted with low melting agarose. Images were acquired with a Leica STELLARIS 5 WLL confocal microscope (Wetzlar, Germany) with 495 nm (SYTO9) and 549 nm (PI) lines. Z-stacks (Z = 10 images collected) were acquired using 40× magnification at 1024 × 1024 pixels and zoom = 0.75 and 2. The images shown are maximum intensity projections, and orthogonal sections are displayed using LAS X software. Three-dimensional reconstructions were made using the FIJI (ImageJ™) 3D Viewer plugin displayed as volume. The sets of experimental combinations were visualized twice. Two z-stacks were taken and analyzed for each combination from different places in the chamber.

### Persister cells induction and treatment

*A. baumannii* was grown in LB broth at 37 °C with agitation. The overnight culture was diluted to OD_595_ = 0.1 in fresh LB broth and supplemented with reline at concentrations of 1×MIC, 3×MIC, 5×MIC, MLE-15 at concentrations of 1×MIC, 3×MIC, 5×MIC, 10×MIC and antibiotic (meropenem) at a concentration of 10×MIC (5 µg/mL; based on preliminary screening tests). This was followed by incubation at 37 °C for 24 h. After 3, 6 and 24 h, the samples were serially diluted and plated onto LB agar. The plates were then incubated at 37 °C for 16 h. The colonies were counted, and the results are expressed as log CFU/mL^53^. The bacteria were plated on LB agar supplemented with meropenem (10×MIC) to exclude the possibility that the colonies originated from spontaneous meropenem-resistant mutants. The colonies that could not grow on meropenem supplemented LB agar were considered persister cells. After that, meropenem-tolerant persister cells were treated with reline or MLE-15 at a concentration of 1×MIC (12.5% v/v and 100 µg/mL, respectively), and incubated at 37 °C for 24 h. After 3, 6 and 24 h, the samples were diluted and plated onto LB agar. The plates were incubated at 37 °C for 16 h. The colonies were counted, and the results were expressed as log CFU/mL.

### Statistical analysis

All the experiments were duplicated in three independent trials (*n* = 6). Bacterial counts and biofilm densities were expressed as log CFU/mL. Significant differences between the treated and control samples were determined via one-way analysis of variance (ANOVA). All the statistical analyses were performed using Statistica software ver. 13.3 (StatSoft Inc., Tulsa, OK, USA). Differences were considered significant at a level of probability *p* < 0.05.

## Results and discussion

### Protein model prediction

**Fig. 3:**
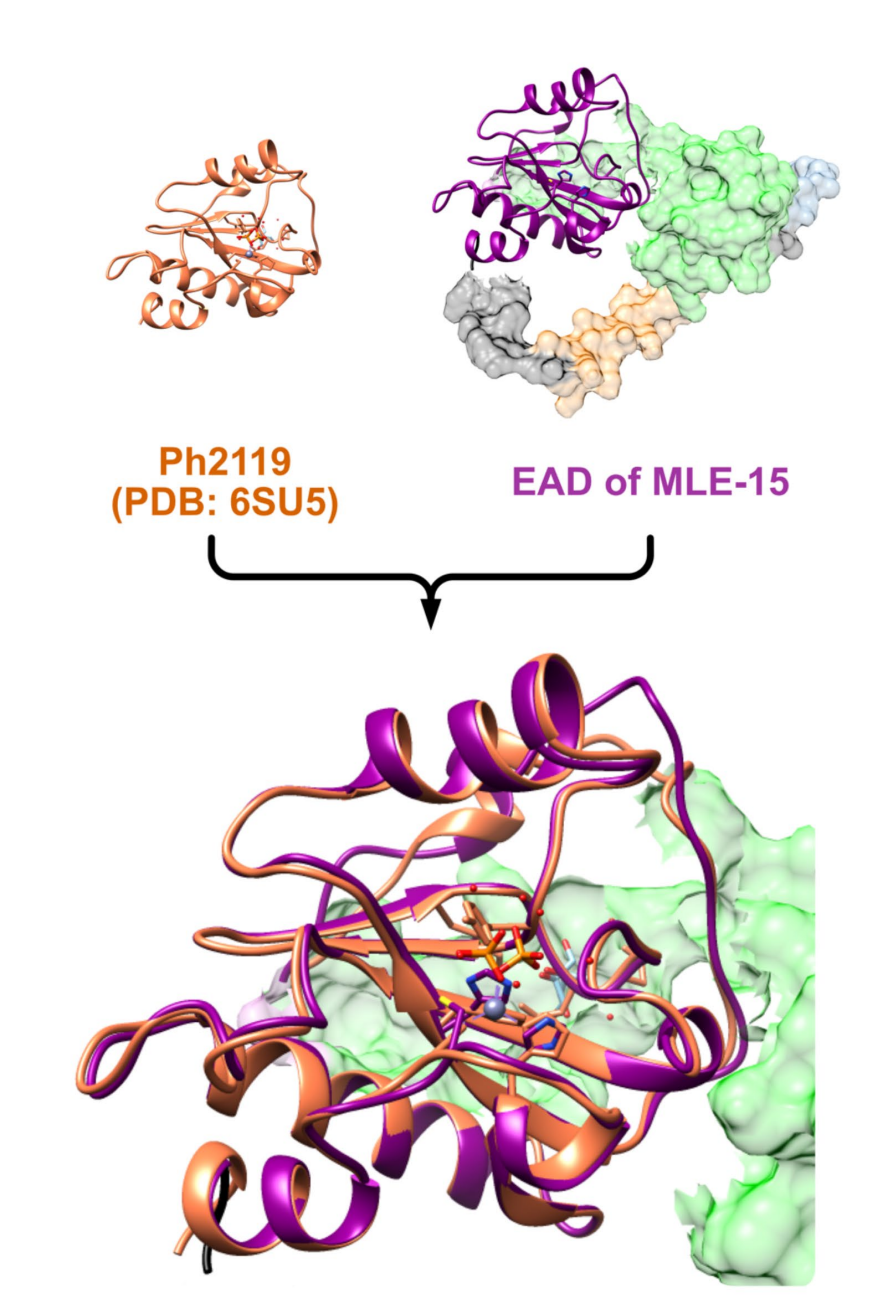
Superposition of EAD of MLE-15 (represented in dark magenta color) and catalytic domain of Ph2119 endolysin (represented in orange color). Model of MLE-15 was obtained with OmegaFold^43^ and was superimposed at the EAD position with Ph2119 (PDB: 6SU5). The colors of the remaining parts of the MLE-15 enzyme are cecropin A - light orange, linker - blue, CBD - green, C-terminal His-tag - black. Superposition was performed with Chimera 1.16^48^ and visualization with use of Affinity Designer (https://affinity.serif.com/en-us/designer/).

The composition of MLE-15 starts with cecropin A, which is connected via a linker to CBD and then to EAD. Bioinformatics was utilized to visualize the tertiary structure of MLE-15 and facilitate understanding of the protein’s overall morphology and the relative positions of its domains. The analysis began with the superposition of models of MLE-15 obtained through various prediction algorithms, including ESMFold, OmegaFold, MODELLER, Phyre2, DeepFold, and AlphaFold2 as well as the known crystallographic structure of EAD domain Ph2119 (PDB: 6SU5) **(**Fig. 3**).**

The model predicted by DeepFold was rejected first, because it did not adequately cover Ph2119 and improperly folded the protein, resulting in domains being scattered in a non-globular and highly improbable manner. The Phyre2 model was dismissed as its CBD domain did not align with the other models. While modeling with MODELLER, no template was found for Met1-Ala53 residues in the sequence of MLE-15. Hence, the model obtained with OmegaFold was used as a template by MODELLER instead of covering the missing template. However, this model was also rejected as it merely duplicated the OmegaFold model, rather than creating an original structure. The AlphaFold2 model, which suggested a loop structure between Met1 and Gly110, was discarded due to the expectation of an α-helical structure between Gly2 and Lys37, characteristic of cecropin A^54^. Subsequently, the investigation focused on cecropin A, the OMP component of MLE-15. ESMFold and OmegaFold algorithms predicted tertiary structure of the N-terminal part of the protein with high probability.

These two models were then analyzed using the Foldseek tool in search of similar structures in protein’s databases. No structures were identified using the ESMFold model, but the OmegaFold model produced several search hits. The TM-score, which measures topological similarity between protein structures, where 1.0 is the highest score, was the best for cecropin A2 (UniProt: Q963B0) with a score of 0.774. Other notable scores included cecropin B1 (UniProt: Q86PR5) at 0.73149 and cecropin C (UniProt: Q8MUF3) at 0.603777, all found in the SWISSPROT database. The only structure found in the RCSB Protein Data Bank was the H5 hemagglutinin mutant Y161A from the A/Viet Nam/1203/2004 H5N1 influenza virus, with similarities in chains B, D, and F. The highest TM-score was 0.67043 in chain D (PDB: 6E7H).

**Fig. 4:**
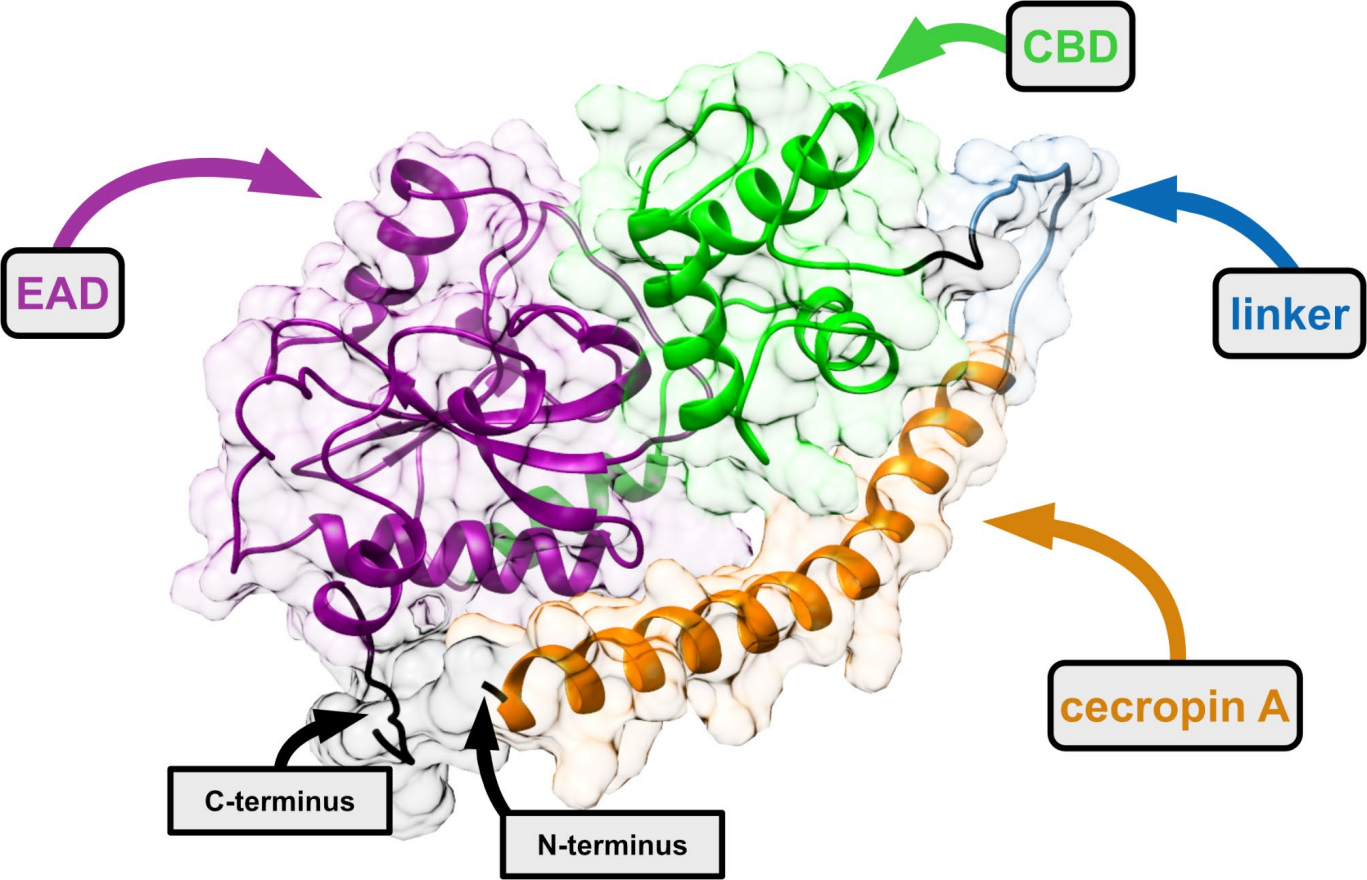
Structural model of MLE-15 generated with OmegaFold^43^. The enzyme consists of four building blocks antimicrobial peptide cecropin A from *Aedes aegypti* (orange), 14 amino acid flexible linker (blue), cell wall binding domain (CBD) of endolysin 201phi2-1 from *Pseudomonas* phage 201phi2-1 (green), and enzymatically active domain (EAD) of Ph2119 endolysin from *Thermus* phage Ph2119 (dark magenta). His-tag at the C-terminus and the N-terminus are depicted in black. Surface transparency visible in the background of the model is 80%. Figure was prepared with Affinity Designer software.

After comparing all models, the tertiary structure constructed using OmegaFold was chosen as the most accurate (Fig. 4). To our knowledge, this is the first protein model representing the structure of the modular enzyme consisting of four building blocks, obtained using the VersaTile methodology.

### Overproduction, homogeneity and thermal stability of the MLE-15

**Fig. 5:**
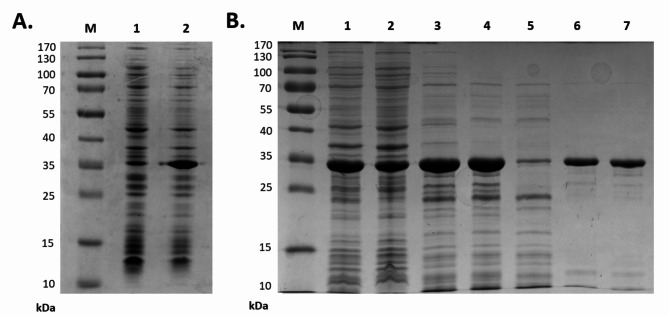
Overproduction (**A**) and purification (**B**) of MLE-15. The results of overproduction, where: M – marker proteins, lane 1 – sample before induction, and lane 2 – sample after induction. The results of subsequent purification steps, where: M – marker proteins, lane 1 – total protein (lysate after sonication), lane 2 – pellet, lane 3 – supernatant, lane 4 – protein after heating, washing with increasing imidazole concentration in NPI buffer lane 5 − 10 mM, lane 6 − 20 mM, and lane 7 − 150 mM. Uncropped images of gels are shown in Figure S2.

MLE-15 was overproduced in the *E. coli* system (Fig. 5A) and purified via metal affinity chromatography through its C-terminal His-tag (Fig. 5B). Analysis of the subsequent purification steps visualized by SDS-PAGE showed that, after purification and dialysis against storage buffer (20 mM HEPES-NaOH, pH 7.4, 50% glycerol), MLE-15 migrated as a single band according to its predicted molecular mass of 33.43 kDa (Fig. 5B, **lane 7**). The final yield of recombinant protein purification was 10.5 mg/L of bacterial culture.

**Fig. 6:**
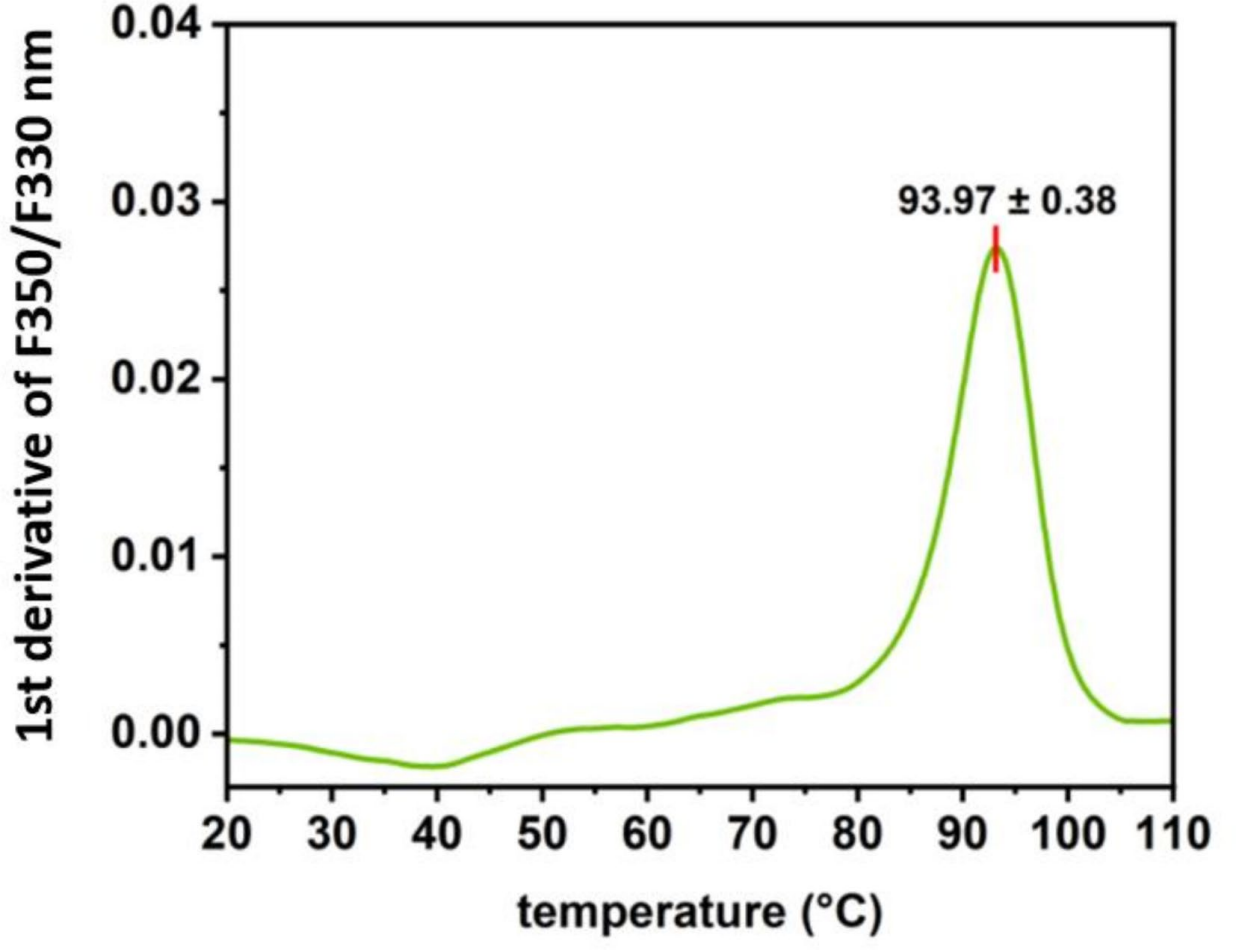
Thermal denaturation profile of MLE-15. The inflection point corresponds to the melting temperature (Tm) value.

MLE-15 was present in a soluble protein fraction after heat treatment of the bacterial lysate, which proved the stability of the tested protein (Fig. 5, **lane 4**). To investigate the thermal stability of MLE-15, we implemented nanoDSF, which allowed us to determine the protein melting temperature at which 50% of the protein is in its unfolded form (Fig. 6). The melting temperature of MLE-15 was determined to be 93.97 ± 0.38 °C, which indicated that the enzyme is extremely stable at high temperatures, which is a desirable feature in the production of enzymes of potential use in the biotech industry.

### Growth inhibitory effect of MLE-15, reline, and their combination

MIC values for MLE-15 and reline were determined against Gram-positive and Gram-negative bacteria (Table 2). The MIC values for MLE-15 were consistently 100 µg/mL across all tested strains. In contrast, the MIC values for reline varied by strain, ranging from 6.25 to 25% v/v. The lowest value of MIC for reline (6.25% v/v) was observed for *(A) baumannii* CRAB, the highest (25% v/v) for *(B) subtilis*, while the MIC for the remaining strains was 12.5% v/v. The MIC values of reline were also tested in comparison to MICs of its parent substances (Supplementary Table S1). All MIC values ​​for reline were lower than the MICs of the constituent substances, therefore reline was selected for further testing. The effect of the combination of MLE-15 with reline was estimated based on fractional inhibitory concentrations (ƩFIC). The combination of MLE-15 with reline at its sub-MIC concentration (4.5% v/v) reduced the MIC of MLE-15 from 100 µg/mL to 3.15–50 µg/mL depending on the tested stain. However, for most strains, the ΣFIC value indicated an additive effect (0.5 < ΣFIC ≤ 1), with synergy observed only for *(A) baumannii* RUH134 and *(B) subtilis* (ΣFIC ≤ 0.5). The MIC values of MLE-15 were reduced from 100 µg/mL to 6.25 µg/mL for *(A) baumannii* RUH134 and 12.5 µg/mL for *(B) subtilis*. This data demonstrated that the presence of reline reduced the MIC of MLE-15 while maintaining its inhibitory effect on bacterial growth, suggesting that reline could potentially enhance the antibacterial properties of other substances.

**Table 2:** MIC values of MLE-15, reline, and MLE-15 in the presence of reline (sub-MIC, 4.5% v/v) against selected Gram-positive and Gram-negative bacterial strains. The effect of interaction was estimated based on fractional inhibitory concentrations (ƩFIC) as follows: ƩFIC ≤ 0.5 (synergistic), 0.5 < ƩFIC ≤ 1 (additive), 1 < ƩFIC ≤ 4 (indifferent) and ƩFIC > 4 (antagonistic).

Strain	MLE-15	Reline [% v/v]	MLE-15 + reline*		
	[µg/mL]		MLE-15 + reline	ƩFIC	Interaction
*P. aeruginosa* PAO1	100	12.5	50	0.88	additive effect
*P. fluorescens* DSM 50,090	100	12.5	50	0.88	additive effect
*K. pneumoniae* Z4	100	12.5	50	0.88	additive effect
*E. coli* P4-7250	100	12.5	50	0.88	additive effect
*A. baumannii* CRAB	100	6.25	3.15	0.80	additive effect
*A. baumannii* RUH134	100	12.5	6.25	0.45	synergism
*S. aureus* Z7	100	12.5	12.5	0.51	additive effect
*E. faecalis* Z9	100	12.5	50	0.88	additive effect
*B. subtilis* ATCC6633	100	25	12.5	0.32	synergism

* sub-MIC 4.5% v/v.

### Bactericidal effect of reline and MLE-15

**Fig. 7:**
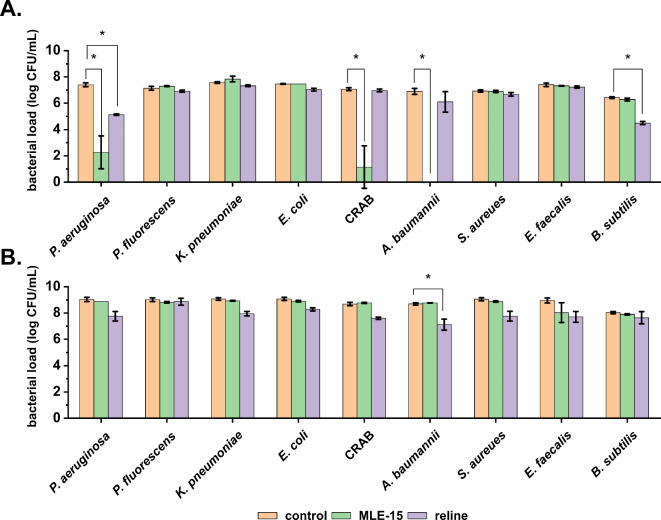
The effect of MLE-15 and reline against Gram-positive and Gram-negative bacteria in planktonic form (**A**) and biofilm (**B**). The results were obtained via the colony counting method after using MLE-15 and reline at concentrations of 1×MIC for 1.5 h for plankton and 2×MIC for 3 h for biofilm. The asterisks indicate statistically significant differences compared to the control (*p* < 0.05). Error bars represent the standard deviation.

Based on the observed MIC values, we assessed the antibacterial activity of MLE-15 and reline against Gram-positive and Gram-negative bacteria in planktonic and biofilm forms (Fig. 7). MLE-15 displayed activity against selected planktonic cells of Gram-negative bacteria, showing complete inhibition of the growth of *A. baumannii* RUH134, and a significant reduction in the number of cells of *(A) baumannii* CRAB (5.92 log units), and *P. aeruginosa* PAO1 (5.12 log units). On the other hand, reline caused significant reduction only in the number of planktonic cells of *P. aeruginosa* (2.26 log units) and *(B) subtilis* (1.84 log units). It should be noted, that the activity of MLE-15, similarly to other modular enzymes, such as 1D10 consisting of four building blocks (peptide, linker, CBD, and EAD), investigated by Gerstmans et al., (2020), may depend on the tonicity of the reaction buffer^18,51^. Our results show that in the absence of NaCl or in hypotonic buffer with 30 mM NaCl, MLE-15 caused eradication of *A. baumannii* RUH134 cells in antibacterial test (≥ 6.40 log units), while 3.19 log reduction of cells counts was observed in the presence of 150 mM NaCl (Supplementary Figure S1). The bactericidal effect of MLE-15 was abolished at 350 mM NaCl, what was also observed in case of 1D10 variant^18^. Therefore, it should be emphasized, that detailed reaction conditions need to be listed while studying activity of modular lytic enzymes. In relation to the biofilms, MLE-15 was ineffective against all strains tested (*p* > 0.05), while reline reduced the number of bacterial cells in all biofilms with a significant reduction of *A. baumannii* RUH134 (1.57 log units).

These findings are consistent with previous literature, where DESs were proven to affect biofilms while not completely eradicating them. For example, in the work of Nystedt et al.^55^, authors investigated three DESs comprising choline chloride and xylitol, choline chloride and glycerol, as well as betaine and sucrose against biofilms of *S. aureus* and *P. aeruginosa*. They found that these DESs reduced biofilm biomass by 27–67% for *S. aureus* and 34–49% for *P. aeruginosa*. In another study by Nava-Ocampo et al.^56^, the authors examined two DESs composed of betaine, urea, and water, as well as lactic acid, glucose, and water, against aerobic granular sludge imitating biofilm. The investigated DESs dissolved up to 70% of the extracellular matrix components, weakening the biofilm structure and potentially facilitating its eradication. Silva et al.^32^ used DESs based on fatty acids (capric acid, myristic acid, lauric acid, and stearic acid) against biofilms formed by *S. aureus*,* E. coli*, and *C. albicans*, demonstrating that a 10-minute exposure to a DES composed of capric acid and lauric acid resulted in over a 50% reduction in biomass of all the investigated biofilms.

However, merely reducing the number of cells in a biofilm without its complete eradication is insufficient in the fight against biofilms. This is due to possible regeneration of the biofilm, which is likely to cause recurrent infections. Despite this, our results and the previously cited studies indicate that DESs can disrupt biofilm structure. This disruption may facilitate the penetration of the antibacterial substances into the complex structure of the biofilm, resulting in more effective interaction with bacterial cells. Therefore, substances that can disrupt the biofilms’ structure (such as conventional DESs like reline) seem promising as co-drugs with other antimicrobials to enhance their efficacy against biofilms.

**Fig. 8:**
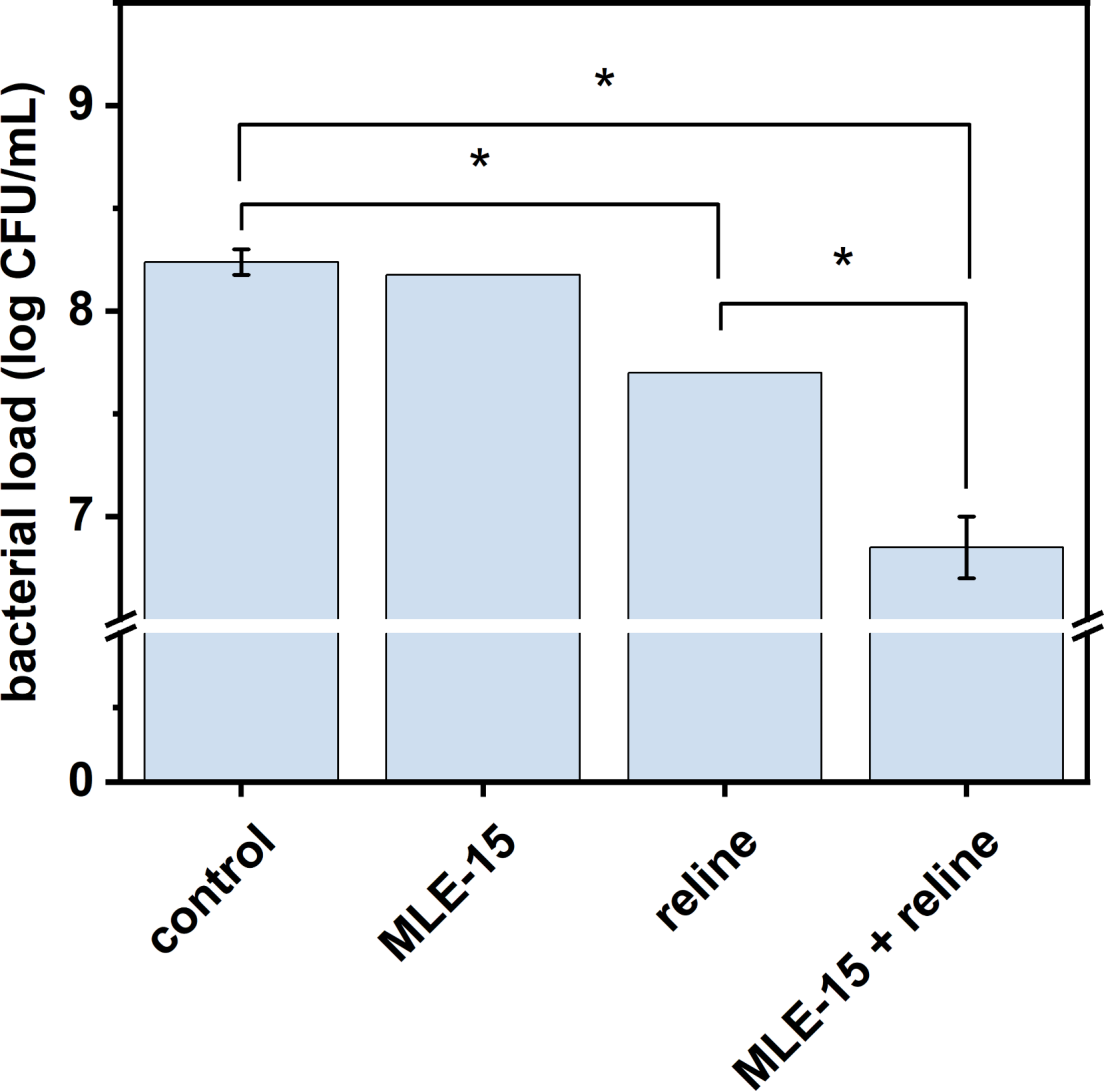
The effect of MLE-15 and reline (2×MIC) and the combination of MLE-15 (1×MIC) with reline (sub-MIC, 4.5% v/v) against matured biofilm of *A. baumannii* RUH134. The results were obtained via the colony counting method after treatment for 3 h. The asterisks indicate statistically significant differences (*p* < 0.05). Error bars represent the standard deviation.

MLE-15 completely inhibited the growth of planktonic cells of *A. baumannii* RUH134 but did not show effectiveness in eradicating biofilms. On the other hand, reline was ineffective against planktonic cells but significantly reduced the bacterial load in biofilms of the same strain. Therefore, we investigated the effects of both antimicrobials against this strain in more detail. First, we assessed the impact of these substances against the matured biofilm (Fig. 8). The results confirmed that MLE-15 was ineffective against biofilm (*p* > 0.05), while reline significantly reduced the number of cells in the biofilm (0.54 log units).

Considering the synergy when MLE-15 was combined with reline, the antibacterial effect of this combination was also examined. The experiment proved that the combination of MLE-15 and reline resulted in a greater reduction (1.39 log units) in the number of cells in the biofilm than when reline was used alone.

To sum up, we proved that MLE-15 at a concentration of 2×MIC was ineffective against *A. baumannii* RUH134 biofilm, while the presence of reline (sub-MIC) significantly reduced the presence of *A. baumannii* RUH134 in the biofilm at a concentration of 1×MIC. Thus, it confirmed again that the presence of reline enhanced the antibacterial activity of MLE-15.

A similar approach, where an external substance was used as a co-drug, was reported in a pioneering study on the assessment of the antibacterial properties of MLE, which was obtained using the VersaTile method^18^. Adding 0.2 mM EDTA – chelator of divalent cations – to lead variant 1D10 reduced its MIC value from 11.8 ± 5.2 to 4.4 ± 3.6 µg/mL in tests on *A. baumannii*. Moreover, it was shown that 0.2 mM EDTA slightly increased the antibacterial effect against *A. baumannii* strains in 90% human serum (reduction from 2.4 to 2.7 to 2.7–3.1 log units). The use of substances that enhance the antibacterial effect of lysins is known in the literature and has been reported as early as 2012^16,57,58^. It is common to use EDTA or weak organic acids as outer membrane permeabilizers, allowing lysins to reach and degrade the peptidoglycan layer. For instance, Oliveira et al.^57^ used EDTA and weak organic acids (malic and citric) to evaluate the antibacterial properties of endolysin Lys68. Authors found that supplementation of Lys68 with 0.2 mM EDTA displayed an antibacterial effect against *Pseudomonas* strains, while the presence of 5 mM malic and 2 mM citric acid broadened the antibacterial effect of Lys68 to *Salmonella* Typhimurium. Moreover, it was demonstrated that combinations malic acid/Lys68 and citric acid/Lys68 reduced the number of *Salmonella* cells by 3–5 log units compared to control samples treated with acids alone^57^. Therefore, our research on combining the antibacterial action of MLE-15 and reline fits existing trends. This approach is also innovative due to the co-action of MLE with deep eutectic solvents represented here by reline, which has not been studied in this context so far. Moreover, our study indicates a possible mechanism of action combining MLE-15 with reline. We showed that MLE-15 had an antibacterial effect on planktonic cells of *A. baumannii* RUH134 at a concentration of 1×MIC, while using up to twice the concentration did not affect the biofilm. However, adding reline at a sub-MIC concentration allowed for a significant reduction of cells in the biofilm when combined with MLE-15.

**Fig. 9:**
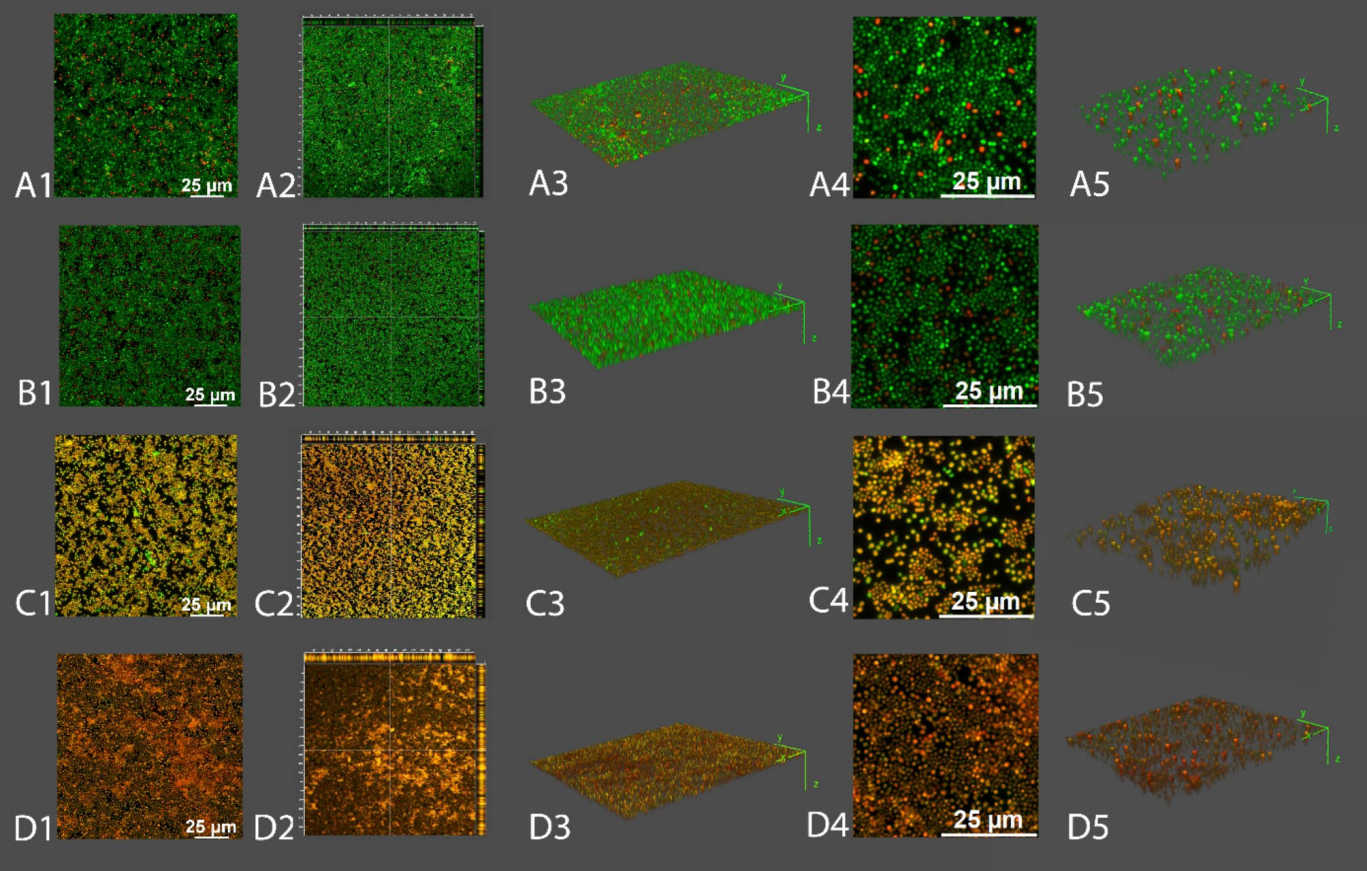
Images of LIVE-DEAD staining for the biofilm of *A. baumannii* RUH134 treated with MLE-15 (2×MIC), reline (2×MIC) or the combination of MLE-15 (1×MIC) and reline (sub-MIC, 4.5% v/v). The 0.85% saline was used as a control. Representative images of the control (**A**), MLE-15 (**B**), reline (**C**), and combination of MLE-15 with reline (**D**). A1, B1, C1, and D1 are maximum intensity projections taken from z-stacks. A2, B2, C2, and D2 are orthogonal sections displayed at 40× magnification. A3, B3, C3, and D3 are 3D reconstructions from A1, B1, C1, and D1, respectively. A4, B4, C4, and D4 are magnifications from A1, B1, C1, and D1, respectively. A5, B5, C5, and D5 are 3D reconstructions from A4, B4, C4, and D4, respectively. The orthogonal sections are displayed using LAS X software (https://www.leica-microsystems.com/products/microscope-software/p/leica-las-x-ls/). Three-dimensional reconstructions were made using the FIJI (ImageJ™) 3D Viewer plugin displayed as volume (https://imagej.net/ij/index.html).

Considering observations mentioned above and the lack of antibacterial effect of MLE-15 alone on the biofilms, one can assume that the activity of reline is involved in the dissolution of the extracellular matrix of the biofilm. Thus, we suspect that reline disrupted the EPS, facilitating the penetration of MLE-15 into the biofilm structure which allowed MLE-15 to act directly on bacterial cells. These observations were supported by microscopy images obtained while implementing LIVE/DEAD staining on the biofilms (Fig. 9). The untreated biofilm showed that *A. baumannii* RUH134 formed a densely-packed biofilm, mostly labeled with SYTO 9 (green cells), with few scattered, damaged cells labeled with PI (red cells), indicating high cell viability within the biofilm (Fig. 9A1). The biofilm treated with MLE-15 displayed a visual effect similar to the untreated control, which showed the lack of activity of MLE-15 against the biofilm of *A. baumannii* RUH134 (Fig. 9B1). On the contrary, biofilms treated with reline (Fig. 9C1) appeared yellowish-orange, while the combination of MLE-15 and reline (Fig. 9D1) resulted in red-stained spots. 3-dimensional analysis of the biofilms indicated that the control (Fig. 9A3, 9A5) and MLE-15-treated biofilms (Fig. 9B3, 9B5) were thicker than biofilms treated with reline (Fig. 9C3, 9C5) or the combination of MLE-15 with reline (Fig. 9D3, 9D5). Furthermore, structural analysis showed very few spaces devoid of cells in the control (Fig. 9A2, 9A4) and MLE-15-treated-biofilm (Fig. 9B2, 9B4), while reline-treated biofilm (Fig. 9C2, 9C4) and biofilm treated with a combination of both (Fig. 9D2, 9D4) resulted in the formation of holes and large areas without visible accumulated biomass. The observations mentioned above confirmed that MLE-15 alone affected neither the viability of *A. baumannii* RUH134 cells in the biofilm nor the structure of the biofilm. On the contrary, using reline alone caused a visible disintegration of the biofilm structure resulting in the cells stained yellowish-orange, indicating a specific effect on the physiology of bacterial cells. In turn, the use of reline combined with MLE-15 revealed damage to the biofilm structure by reline, which enabled the penetration of MLE-15 into the biofilm and a direct effect on the damage of the cytoplasmic membranes of bacterial cells which allowed the penetration of PI into the cells. Presumably, reline alone requires more prolonged action time to affect bacterial cells substantially. However, from the medical point-of-view, it seems more reasonable to use reline with another antibacterial substance, such as MLE-15, than to extend the time needed to inactivate bacterial pathogens.

Previously-mentioned studies on assessing the impact of DESs on biofilms^6,32,55^ also indicated that DESs disrupted the biofilm structure. Our proposed combination of reline with MLE-15 seems to be important in the context of an effective fight against biofilms, which may give more satisfactory results than DESs alone.

### Treatment and induction of emergence of persister cells

**Fig. 10:**
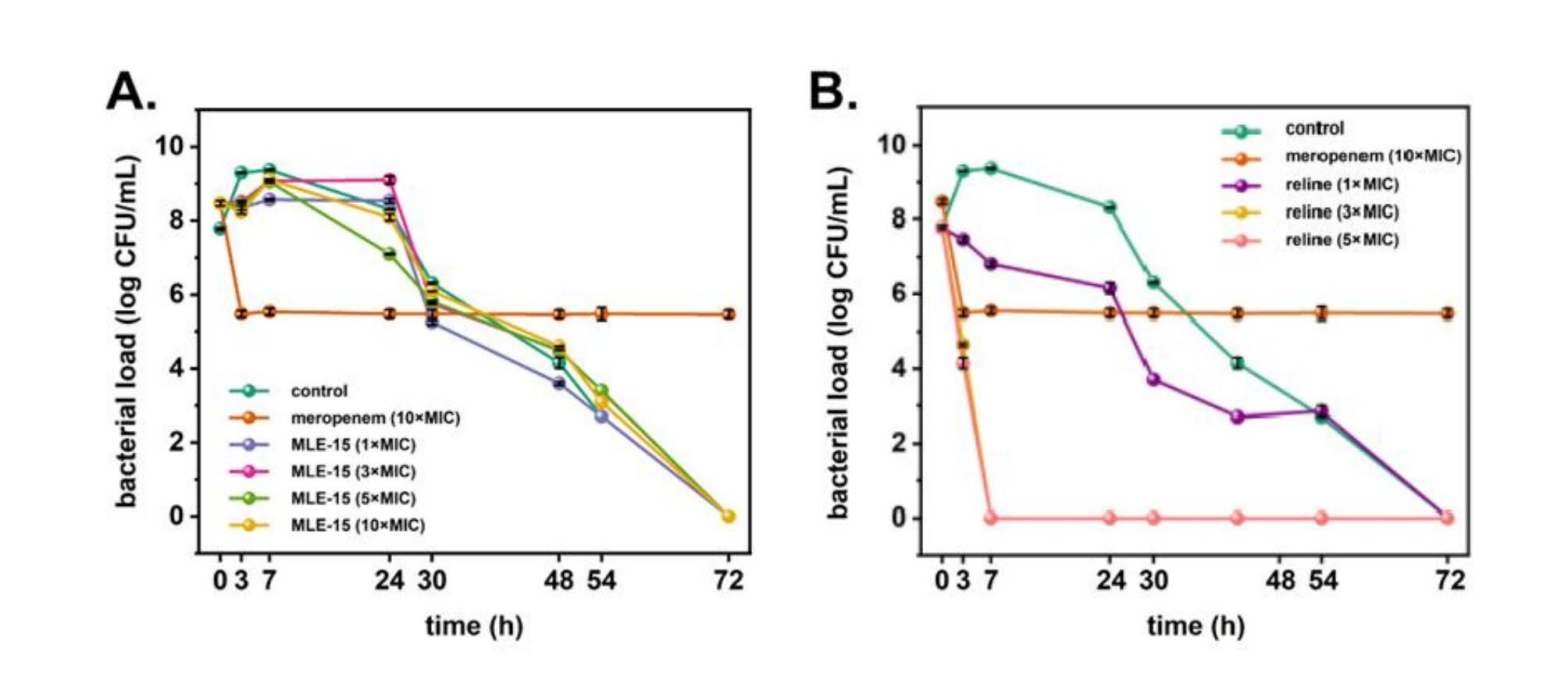
The effect of MLE-15 at the concentrations of 1×, 3×, 5× and 10×MIC (**A**) and reline at concentrations of 1×, 3× and 5×MIC (**B**) compared to that of the control (no treatment) and antibiotic (meropenem) at a concentration of 10×MIC on the induction of persister cells of *A. baumannii* RUH134. Error bars represent the standard deviation.

In addition to the biofilms, persister cells are the leading cause of recurring bacterial infections and pose a formidable challenge to modern medicine. Therefore, the next step in our studies was to investigate whether MLE-15 and reline cause the emergence of persisters of *A. baumannii* RUH134. Our study showed that MLE-15 (Fig. 10A) used at concentrations of 1×, 3×, 5× and 10×MIC as well as reline (Fig. 10B) used at concentration of 1×, 3× and 5×MIC did not cause to emergence of persister cells. Moreover, it was found that MLE-15, regardless of concentration, caused gradual cell death over time until complete eradication 72 h after exposure (Fig. 10A). In turn, reline at a concentration of 3× and 5×MIC caused complete eradication of *A. baumannii* RUH134 after only 7 h of treatment (Fig. 10B). These results are extremely valuable due to the eradication, not just the reduction of *A. baumannii* RUH134 cells and the lack of tolerance to these antimicrobials.

**Fig. 11:**
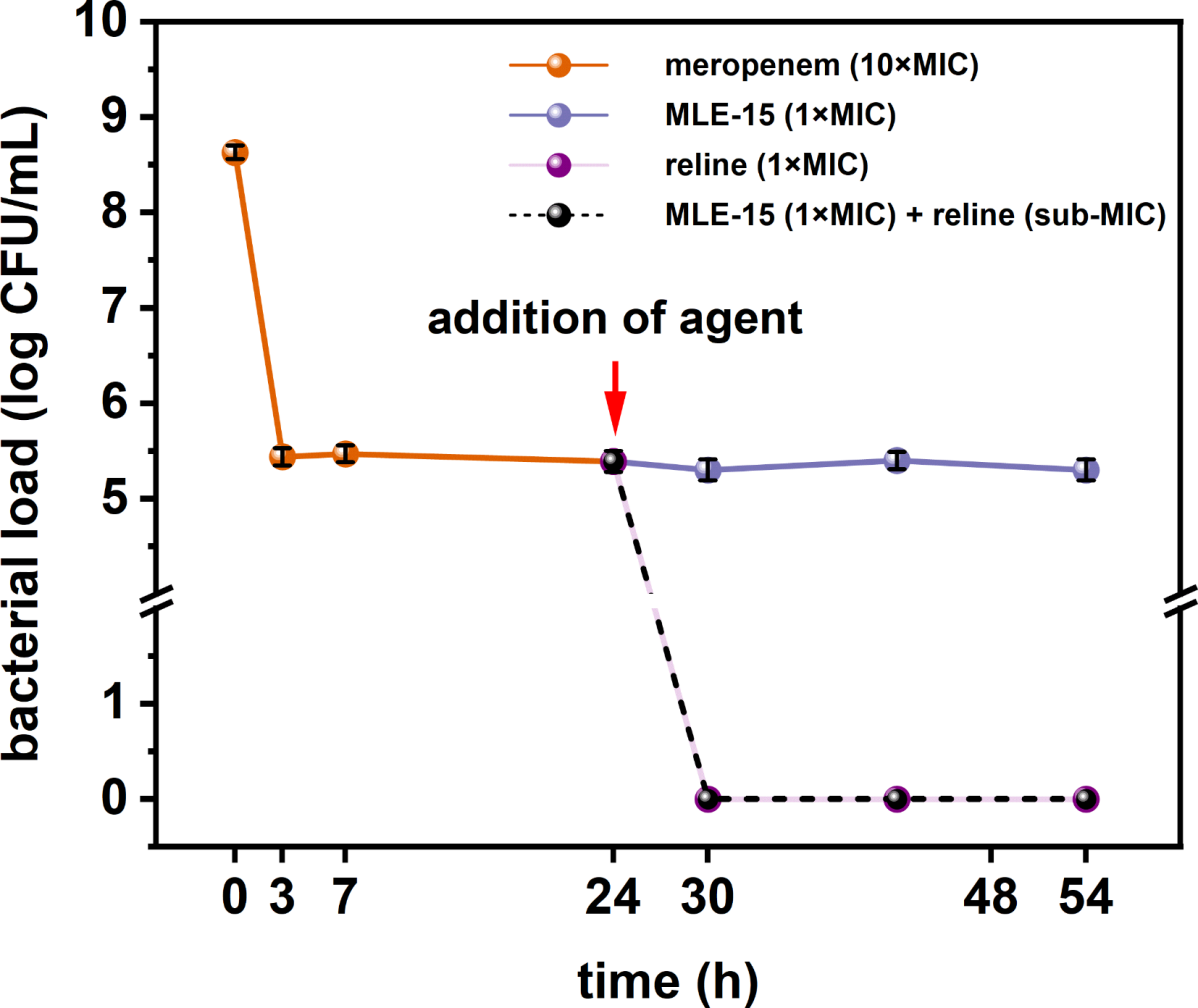
The effect of MLE-15 (1×MIC), reline (1×MIC) and the combination of MLE-15 (1×MIC) with reline (sub-MIC) on meropenem-tolerant persister cells of *A. baumannii* RUH134. Error bars represent the standard deviation.

Finally, we assessed whether MLE-15, reline, and their combination could combat persister cells of *A. baumannii* RUH134 induced using an antibiotic – meropenem (10×MIC) (Fig. 11). Meropenem-induced persister cells were treated with MLE-15 (1×MIC), reline (1×MIC), and their combination (MLE-15–1×MIC and reline – sub-MIC). After the addition of MLE-15, the number of the cells remained stable at the level of approx. 5 log CFU/mL for a total of 24 h, which indicated the lack of the activity of MLE-15 against persisters. In turn, reline, and its combination with MLE-15 eradicated persister cells in just 3 h after treatment, and this effect was maintained in the following hours, which indicated strong activity against persister cells.

These observations are valuable due to the global AMR crisis. Antibiotic-tolerant persister cells are one of the reasons for the overuse of antibiotics. Therefore, our results show that reline and its combination with MLE-15 completely eradicated meropenem-induced persister cells and provided hope for effective antibacterial therapy in the near future.

## Conclusion

In the current global crisis related to antimicrobial resistance, it is imperative to search for novel and innovative solutions to counteract bacterial infections effectively. In this study, we investigated the antibacterial activity of MLE-15, a modular lytic enzyme based on an enzymatically active domain from thermostable endolysin Ph2119, obtained with the VersaTile method. We also examined reline, a natural deep eutectic solvent and their combination. Our result showed that MLE-15 completely inhibited the growth of planktonic cells of extensively resistant strain *A. baumannii* RUH134, while reline significantly reduced the number of *A. baumannii* RUH134 cells in the biofilm. Moreover, both antimicrobials significantly reduced the number of cells in fully formed biofilm. This finding suggests that the activity of MLE-15, which was ineffective against biofilm when used alone, was enhanced in the presence of reline. It appears that reline disrupts extracellular matrix and destabilizes the biofilm structure. These findings were confirmed by microscopic analysis, which demonstrated that reline enhanced the antibacterial properties of MLE-15, indicating that combining MLE-15 with reline is a promising strategy for combating biofilms. Additionally, our study showed that MLE-15 and reline did not cause the emergence of persister cells, and reline alone or in combination with MLE-15 eradicated persister cells induced in the presence of meropenem. These findings suggest that reline, through its action on the outer layers and destabilizing function, acts as a permeability enhancer for MLE-15, enabling it to act directly on bacterial cells. The complete eradication of meropenem-persister cells of this strain highlights the potential of combining MLE-15 with reline in the fight against bacterial infections and antibiotic-resistant strains.

Endolysins, i.e. lytic enzymes of phage origin (e.g. MLE-15 created by VersaTile methodology) are currently facing great interest in the context of the therapy of bacterial infections (e.g. LysRODI against *S. aureus*^59^, Ph28 against *S. epidermidis*^60^, ORF28 gene product against *E. faecalis*^61^). There is also no shortage of advanced clinical or preclinical trials on the use of endolysins to combat bacterial infections^62,63^. Therefore, the issue of lytic enzymes in therapy is absolutely justified and necessary. However, most endolysins are tested against Gram-positive bacteria, because their effectiveness against Gram-negative bacteria is limited by the presence of an additional cover – the outer membrane. Consequently, studies like ours are a valuable contribution in research on combating Gram-negative bacteria.

In turn, DESs are known in biomedicine as treatment (e.g. wound healing^64^), antimicrobial (e.g. effect against *Escherichia coli*, *Proteus mirabilis*, *Salmonella typhimurium*, *Pseudomonas aeruginosa* and *Staphylococcus aureus*^65^) or tool for increasing the antibacterial properties of other therapeutics (e.g. increase the effects of clindamycin in in vivo tests in mice^30^). Nevertheless, our work is the first to indicate an increase in the antibacterial effect of lytic enzymes in the presence of DES (reline). Using a combination of two or more agents to obtain the desired antibacterial effect is a popular strategy in medicine today. Therefore, the research we propose meets current trends and perfectly combines both innovative antibacterial approaches - the use of lytic enzyme and DES. The combination of these two antimicrobials, which are currently being intensively studied, may constitute a real alternative to conventional antibiotics.

## Electronic supplementary material

Below is the link to the electronic supplementary material.

Supplementary Material 1

## Data Availability

The raw files related to this study are available under CC BY - Creative Commons 4.0 License and can be found in the RepOD repository (https://doi.org/10.18150/OWFIIK).

## References

[1] Murray, C. J. et al. Feb., Global burden of bacterial antimicrobial resistance in 2019: a systematic analysis, *The Lancet*, vol. 399, no. 10325, pp. 629–655, (2022). 10.1016/S0140-6736(21)02724-0 10.1016/S0140-6736(21)02724-0 PMC8841637 35065702

[2] Huemer, M., Shambat, S. M., Brugger, S. D. & Zinkernagel, A. S. Antibiotic resistance and persistence—Implications for human health and treatment perspectives, *EMBO Rep*, vol. 21, no. 12, p. e51034, Dec. (2020). 10.15252/EMBR.202051034 10.15252/embr.202051034 PMC7726816 33400359

[3] Mulani, M. S., Kamble, E. E., Kumkar, S. N., Tawre, M. S. & Pardesi, K. R. Emerging strategies to combat ESKAPE pathogens in the era of antimicrobial resistance: A review, *Front. Microbiol.*, **10**, 403107. 10.3389/FMICB.2019.00539 (2019). 10.3389/fmicb.2019.00539 PMC6452778 30988669

[4] Yu, Z., Tang, J., Khare, T. & Kumar, V. The alarming antimicrobial resistance in ESKAPEE pathogens: can essential oils come to the rescue? *Fitoterapia* **140**10.1016/J.FITOTE.2019.104433 (Jan. 2020). 10.1016/j.fitote.2019.104433 31760066

[5] Szadkowska, M. et al. A Novel cryptic clostridial peptide that kills Bacteria by a cell membrane permeabilization mechanism. *Microbiol. Spectr.* **10**(5). 10.1128/SPECTRUM.01657-22 (2022). 10.1128/spectrum.01657-22 PMC9602519 36094301

[6] Kocot, A. M., Wróblewska, B. & Cabo, M. L. Operational culture conditions determinate benzalkonium chloride resistance in L. monocytogenes-*E. Coli* dual species biofilms. *Int. J. Food Microbiol.* **360**, 109441. 10.1016/J.IJFOODMICRO.2021.109441 (Dec. 2021). 10.1016/j.ijfoodmicro.2021.109441 34717152

[7] Sharma, D., Misba, L. & Khan, A. U. Antibiotics versus biofilm: an emerging battleground in microbial communities, *Antimicrobial Resistance & Infection Control 2019 8:1*, vol. 8, no. 1, pp. 1–10, May (2019). 10.1186/S13756-019-0533-3 10.1186/s13756-019-0533-3 PMC6524306 31131107

[8] Abebe, G. M. The role of bacterial biofilm in Antibiotic Resistance and Food Contamination. *Int. J. Microbiol.* **2020**10.1155/2020/1705814 (2020). 10.1155/2020/1705814 PMC7468660 32908520

[9] Berkvens, A., Chauhan, P. & Bruggeman, F. J. Integrative biology of persister cell formation: molecular circuitry, phenotypic diversification and fitness effects. *J. R Soc. Interface*. **19**(194). 10.1098/RSIF.2022.0129 (2022). 10.1098/rsif.2022.0129 PMC9470271 36099930

[10] Balaban, N. Q. et al. Definitions and guidelines for research on antibiotic persistence. *Nature Rev. Microbiol.*, **17**(7), 441–448. 10.1038/s41579-019-0196-3 (2019). 10.1038/s41579-019-0196-3 PMC7136161 30980069

[11] Levin-Reisman, I. et al. Antibiotic tolerance facilitates the evolution of resistance. *Science*. **355**(6327), 826–830. 10.1126/SCIENCE.AAJ2191 (2017). 10.1126/science.aaj2191 28183996

[12] Lebeaux, D., Ghigo, J. M. & Beloin, C. Biofilm-Related Infections: Bridging the Gap between Clinical Management and Fundamental Aspects of Recalcitrance toward Antibiotics, *Microbiol. Mol. Biol. Rev.* **78**(3), 510 (2014). 10.1128/MMBR.00013-14 (2014). 10.1128/MMBR.00013-14 PMC4187679 25184564

[13] Weber, G., Buhren, B., Schrumpf, H., Wohlrab, J. & Gerber, P. Therapeutic enzymes: function and clinical implications., *Adv Exp Med Biol*, vol. 1148, pp. 233–252, Accessed: Jun. 13, 2024. [Online]. Available: http://link.springer.com/ (2019). 10.1007/978-981-13-7709-9

[14] Danis-Wlodarczyk, K. M., Wozniak, D. J. & Abedon, S. T. Treating Bacterial Infections with Bacteriophage-Based Enzybiotics: In Vitro, In Vivo and Clinical Application, *Antibiotics 2021, Vol. 10, Page 1497*, vol. 10, no. 12, p. 1497, Dec. (2021). 10.3390/ANTIBIOTICS10121497 10.3390/antibiotics10121497 PMC8698926 34943709

[15] Schmelcher, M., Donovan, D. M. & Loessner, M. J. Bacteriophage endolysins as novel antimicrobials. *Fut. Microbiol.* **7**, (10), 1147–1171. 10.2217/FMB.12.97 (2012). 10.2217/fmb.12.97 PMC3563964 23030422

[16] Plotka, M., Kapusta, M., Dorawa, S., Kaczorowska, A. K. & Kaczorowski, T. Ts2631 Endolysin from the Extremophilic *Thermus scotoductus* Bacteriophage vB_Tsc2631 as an Antimicrobial Agent against Gram-Negative Multidrug-Resistant Bacteria, *Viruses 2019, Vol. 11, Page 657*, vol. 11, no. 7, p. 657, Jul. (2019). 10.3390/V11070657 10.3390/v11070657 PMC6669862 31323845

[17] Jeong, T. H., Hong, H. W., Kim, M. S., Song, M. & Myung, H. Characterization of three different endolysins effective against gram-negative bacteria. *Viruses*, **15**(3), 679, 10.3390/V15030679 (2023). 10.3390/v15030679 PMC10053066 36992387

[18] Gerstmans, H. et al. A VersaTile-driven platform for rapid hit-to-lead development of engineered lysins. *Sci. Adv.* **6**(23). 10.1126/SCIADV.AAZ1136 (2020). 10.1126/sciadv.aaz1136 PMC7269649 32537492

[19] Huan, Y., Kong, Q., Mou, H. & Yi, H. Antimicrobial peptides: classification, design, application and research progress in multiple fields. *Front. Microbiol.* **11**, 582779. 10.3389/FMICB.2020.582779 (2020). 10.3389/fmicb.2020.582779 PMC7596191 33178164

[20] Mazurkiewicz-Pisarek, A., Baran, J. & Ciach, T. Antimicrobial peptides: challenging Journey to the Pharmaceutical, Biomedical, and Cosmeceutical Use. *Int. J. Mol. Sci.*. **24**(10), 9031. 10.3390/IJMS24109031 (2023). 10.3390/ijms24109031 PMC10219530 37240379

[21] Mahlapuu, M., Håkansson, J., Ringstad, L. & Björn, C. Antimicrobial Peptides: An Emerging Category of Therapeutic Agents, *Front Cell Infect Microbiol*. **6**, 194. 10.3389/FCIMB.2016.00194 (2016). 10.3389/fcimb.2016.00194 PMC5186781 28083516

[22] Henriques, S. T., Melo, M. N. & Castanho, M. A. R. B. Cell-penetrating peptides and antimicrobial peptides: how different are they? *Biochem. J.* **399**(1), 1–7. 10.1042/BJ20061100 (2006) 10.1042/BJ20061100 PMC1570158 16956326

[23] Plotka, M. et al. Novel highly thermostable endolysin from *Thermus scotoductus* MAT2119 bacteriophage Ph2119 with amino acid sequence similarity to eukaryotic peptidoglycan recognition proteins. *Appl. Environ. Microbiol.* **80** (3), 886–895. 10.1128/AEM.03074-13 (2014). 10.1128/AEM.03074-13 PMC3911187 24271162

[24] Siddiqui, R. et al. The increasing importance of novel deep eutectic solvents as potential effective antimicrobials and other medicinal properties. *World J Microbiol Biotechnol*, **39**, (12), 1–10. 10.1007/S11274-023-03760-8 (2023). 10.1007/s11274-023-03760-8 37792153

[25] Thapa, R. K., Kim, J. O. & Kim, J. Antimicrobial strategies for topical biofilm-based wound infections: past, present, and future. *Journal of Pharmaceutical Investigation*, **53**(5), 627–641. 10.1007/S40005-023-00628-9 (2023).

[26] Svigelj, R., Dossi, N., Grazioli, C. & Toniolo, R. Deep Eutectic solvents (DESs) and their application in Biosensor Development. *Sens. 2021*. **21, Page 4263, 21**, (13), 4263. 10.3390/S21134263 (Jun. 2021). 10.3390/s21134263 PMC8271379 34206344

[27] Tiecco, M. et al. Advances in the development of novel green liquids: thymol/water, thymol/urea and thymol/phenylacetic acid as innovative hydrophobic natural deep eutectic solvents. *J. Mol. Liq*. **364**, 120043. 10.1016/J.MOLLIQ.2022.120043 (Oct. 2022).

[28] Pereira, C. V. et al. Unveil the Anticancer potential of Limomene Based Therapeutic Deep Eutectic solvents. *Sci. Rep.* **9** (1). 10.1038/S41598-019-51472-7 (Dec. 2019). 10.1038/s41598-019-51472-7 PMC6797721 31624310

[29] Lu, C., Cao, J., Wang, N. & Su, E. Significantly improving the solubility of non-steroidal anti-inflammatory drugs in deep eutectic solvents for potential non-aqueous liquid administration. *Medchemcomm* **7** (5), 955–959. 10.1039/C5MD00551E (May 2016).

[30] Zakrewsky, M. et al. Jun., Choline and Geranate Deep Eutectic Solvent as a Broad-Spectrum Antiseptic Agent for Preventive and Therapeutic Applications, *Adv Healthc Mater*, vol. 5, no. 11, pp. 1282–1289, (2016). 10.1002/ADHM.201600086 10.1002/adhm.201600086 26959835

[31] Akbar, N. et al. Antimicrobial activity of Novel Deep Eutectic solvents. *Sci. Pharm.* **91**(1), 9. 10.3390/SCIPHARM91010009 (2023).

[32] Silva, J. M., Silva, E., Reis, R. L. & Duarte, A. R. C. A closer look in the antimicrobial properties of deep eutectic solvents based on fatty acids. *Sustain. Chem. Pharm.* **14**, 100192. 10.1016/J.SCP.2019.100192 (Dec. 2019).

[33] Angsantikul, P. et al. Ionic liquids and deep Eutectic solvents for enhanced delivery of antibodies in the gastrointestinal tract. *Adv. Funct. Mater.* **31**, 2002912. 10.1002/ADFM.202002912 (Oct. 2021).

[34] Agatemor, C. et al. Choline-Geranate Deep Eutectic Solvent improves Stability and Half-Life of Glucagon-Like Peptide-1. *Adv. Ther. (Weinh)*. **4** (3), 2000180. 10.1002/ADTP.202000180 (Mar. 2021).

[35] Khamoushian, S. et al. Aug., Transdermal Delivery of Insulin Using Combination of Iontophoresis and Deep Eutectic Solvents as Chemical Penetration Enhancers: In Vitro and in Vivo Evaluations. *J Pharm Sci*. **112**(8), 2249–2259. 10.1016/J.XPHS.2023.03.005 (2023). 10.1016/j.xphs.2023.03.005 36921801

[36] Hammond, O. S., Bowron, D. T. & Edler, K. J. Liquid structure of the choline chloride-urea deep eutectic solvent (reline) from neutron diffraction and atomistic modelling. *Green Chem.* **18** (9), 2736–2744. 10.1039/C5GC02914G (May 2016).

[37] Silvestro, L., Weiser, J. N. & Axelsen, P. H. Antibacterial and antimembrane activities of cecropin A in *Escherichia coli*. *Antimicrob. Agents Chemother.* **44**(3), 602–607. 10.1128/AAC.44.3.602-607.2000 (2000). 10.1128/aac.44.3.602-607.2000 PMC89733 10681325

[38] Thomas, J. A. et al. Jul., Characterization of *Pseudomonas chlororaphis* myovirus 201ϕ2 – 1 via genomic sequencing, mass spectrometry, and electron microscopy, *Virology*, vol. 376, no. 2, pp. 330–338, (2008). 10.1016/J.VIROL.2008.04.004 10.1016/j.virol.2008.04.004 PMC2577825 18474389

[39] Jumper, J. et al. Jul., Highly accurate protein structure prediction with AlphaFold, *Nature 2021 596:7873*, vol. 596, no. 7873, pp. 583–589, (2021). 10.1038/s41586-021-03819-2 10.1038/s41586-021-03819-2 PMC8371605 34265844

[40] Lin, Z. et al. Evolutionary-scale prediction of atomic-level protein structure with a language model, *Science *, **379**(6637), 1123–1130. 10.1126/SCIENCE.ADE2574 (2023). 10.1126/science.ade2574 36927031

[41] Pearce, R., Li, Y., Omenn, G. S. & Zhang, Y. Fast and accurate ab initio protein structure prediction using deep learning potentials. *PLoS Comput. Biol.* **18** (9), e1010539. 10.1371/JOURNAL.PCBI.1010539 (Sep. 2022). 10.1371/journal.pcbi.1010539 PMC9518900 36112717

[42] Kelley, L. A., Mezulis, S., Yates, C. M., Wass, M. N. & Sternberg, M. J. E. The Phyre2 web portal for protein modeling, prediction and analysis, *Nature Protocols 2015 10:6*, vol. 10, no. 6, pp. 845–858, May (2015). 10.1038/nprot.2015.053 10.1038/nprot.2015.053 PMC5298202 25950237

[43] Wu, R. et al. Jul., High-resolution de novo structure prediction from primary sequence, *bioRxiv*, p. 2022.07.21.500999, (2022). 10.1101/2022.07.21.500999

[44] Šali, A. & Blundell, T. L. Comparative Protein Modelling by Satisfaction of Spatial Restraints, *J Mol Biol*, vol. 234, no. 3, pp. 779–815, Dec. (1993). 10.1006/JMBI.1993.1626 10.1006/jmbi.1993.1626 8254673

[45] van Kempen, M. et al. May., Fast and accurate protein structure search with Foldseek, *Nature Biotechnology 2023 42:2*, vol. 42, no. 2, pp. 243–246, (2023). 10.1038/s41587-023-01773-0 10.1038/s41587-023-01773-0 PMC10869269 37156916

[46] Heo, L., Park, H. & Seok, C. GalaxyRefine: protein structure refinement driven by side-chain repacking. *Nucleic Acids Res.* **41**, W384–W388. 10.1093/NAR/GKT458 (Jul. 2013). no. W1. 10.1093/nar/gkt458 PMC3692086 23737448

[47] Lee, G. R., Heo, L. & Seok, C. Effective protein model structure refinement by loop modeling and overall relaxation, *Proteins: Structure, Function, and Bioinformatics*, vol. 84, no. S1, pp. 293–301, Sep. (2016). 10.1002/PROT.24858 10.1002/prot.24858 26172288

[48] Pettersen, E. F. et al. Jan., UCSF ChimeraX: Structure visualization for researchers, educators, and developers, *Protein Science*, vol. 30, no. 1, pp. 70–82, (2021). 10.1002/PRO.3943 10.1002/pro.3943 PMC7737788 32881101

[49] Minimum Inhibitory Concentration (MIC) and Minimum Bactericidal Concentration (MBC). Assays Using Broth Microdilution Method. Accessed: Jun. 13, 2024. [Online]. Available: https://www.protocols.io/view/minimum-inhibitory-concentration-mic-and-minimum-b-5qpvo3x6dv4o/v1

[50] Szadkowska, M. et al. Molecular characterization of the PhiKo endolysin from *Thermus thermophilus* HB27 bacteriophage phiKo and its cryptic lytic peptide RAP-29. *Front. Microbiol.* **14**, 1303794. 10.3389/FMICB.2023.1303794 (2023). 10.3389/fmicb.2023.1303794 PMC10836841 38312500

[51] Vázquez, R. et al. You get what you test for: the killing effect of phage lysins is highly dependent on buffer tonicity and ionic strength. *Microb. Biotechnol.* **17** (7), e14513. 10.1111/1751-7915.14513 (Jul. 2024). 10.1111/1751-7915.14513 PMC11222872 38962879

[52] Kocot, A. M. & Olszewska, M. A. Interaction and inactivation of *Listeria* and *Lactobacillus* cells in single and mixed species biofilms exposed to different disinfectants, *J Food Saf*, vol. 39, no. 6, p. e12713, Dec. (2019). 10.1111/JFS.12713

[53] Kuczyńska-Wiśnik, D., Stojowska-Swędrzyńska, K. & Laskowska, E. Intracellular Protective Functions and Therapeutical Potential of Trehalose, *Molecules 2024, Vol. 29, Page 2088*, vol. 29, no. 9, p. May 2024, (2088). 10.3390/MOLECULES29092088 10.3390/molecules29092088 PMC11085779 38731579

[54] Steiner, H. Secondary structure of the cecropins: antibacterial peptides from the moth *Hyalophora cecropia*. *FEBS Lett.* **137** (2), 283–287. 10.1016/0014-5793(82)80368-2 (Jan. 1982). 10.1016/0014-5793(82)80368-2 15768483

[55] Nystedt, H. L. et al. Neutral natural deep eutectic solvents as anti-biofilm agents, *Biofilm*, vol. 5, p. 100114, Dec. (2023). 10.1016/J.BIOFLM.2023.100114 10.1016/j.bioflm.2023.100114 PMC10067762 37020863

[56] Nava-Ocampo, M. F. et al. Natural deep eutectic solvents as biofilm structural breakers. *Water Res.* **201**, 117323. 10.1016/J.WATRES.2021.117323 (Aug. 2021). 10.1016/j.watres.2021.117323 34139511

[57] Oliveira, H. et al. A Thermostable *Salmonella* Phage Endolysin, Lys68, with Broad Bactericidal properties against Gram-negative pathogens in Presence of weak acids. *PLoS One*. **9** (10), e108376. 10.1371/JOURNAL.PONE.0108376 (Oct. 2014). 10.1371/journal.pone.0108376 PMC4188523 25290100

[58] Walmagh, M., Briers, Y., dos Santos, S. B., Azeredo, J. & Lavigne, R. Characterization of modular bacteriophage endolysins from *Myoviridae* Phages OBP, 201φ2 – 1 and PVP-SE1. *PLoS One*. **7** (5), e36991. 10.1371/JOURNAL.PONE.0036991 (May 2012). 10.1371/journal.pone.0036991 PMC3352856 22615864

[59] Portilla, S., Fernández, L., Gutiérrez, D., Rodríguez, A. & García, P. Encapsulation of the Antistaphylococcal Endolysin LysRODI in pH-Sensitive liposomes. *Antibiot. 2020*. **9** (5), 242. 10.3390/ANTIBIOTICS9050242 (May 2020). Page 242. 10.3390/antibiotics9050242 PMC7277728 32397435

[60] Muharram, M. M., Abulhamd, A. T., Aldawsari, M. F., Alqarni, M. H. & Labrou, N. E. Development of *Staphylococcus* Enzybiotics: The Ph28 Gene of *Staphylococcus epidermidis* Phage PH15 Is a Two-Domain Endolysin, *Antibiotics 2020, Vol. 9, Page 148*, vol. 9, no. 4, p. 148, Mar. (2020). 10.3390/ANTIBIOTICS9040148 10.3390/antibiotics9040148 PMC7235722 32235599

[61] Zhang, H. et al. Jul., Bacteriophage φEf11 ORF28 Endolysin, a Multifunctional Lytic Enzyme with Properties Distinct from All Other Identified *Enterococcus faecalis* Phage Endolysins, *Appl Environ Microbiol*, vol. 85, no. 13, pp. 555–574, (2019). 10.1128/AEM.00555-19 10.1128/AEM.00555-19 PMC6581165 30979842

[62] Harhala, M. et al. Safety Studies of Pneumococcal Endolysins Cpl-1 and Pal, *Viruses* Vol. 10, Page 638, vol. 10, no. 11, p. 638, Nov. 2018, (2018). 10.3390/V10110638 10.3390/v10110638 PMC6266847 30445722

[63] Jun, S. Y. et al. Preclinical safety evaluation of intravenously administered SAL200 containing the recombinant phage endolysin SAL-1 as a pharmaceutical ingredient. *Antimicrob. Agents Chemother.* **58** (4), 2084–2088. 10.1128/AAC.02232-13 (2014). 24449776 10.1128/AAC.02232-13 PMC4023757

[64] Silva, J. M. et al. Therapeutic role of Deep Eutectic solvents based on menthol and saturated fatty acids on Wound Healing. *ACS Appl. Bio Mater.* **2**(10), 4346–4355. 10.1021/ACSABM.9B00598 (2019). 10.1021/acsabm.9b00598 PMC6993812 32030369

[65] Radošević, K. et al. Antimicrobial, cytotoxic and antioxidative evaluation of natural deep eutectic solvents. *Environ. Sci. Pollut. Res.* **25**(14), 14188–14196. 10.1007/S11356-018-1669-Z (2018). 10.1007/s11356-018-1669-z 29524174

