# Supplementary material for: Deep eutectic solvent enhances antibacterial activity of a modular lytic enzyme against Acinetobacter baumannii
Source: Sci Rep. 2025 Jan 15;15:2047. doi: 10.1038/s41598-024-80440-z (PMC11735859; doi:10.1038/s41598-024-80440-z)
Supplement: Supplementary file 1 — Supplementary Material 1 [file 41598_2024_80440_MOESM1_ESM.docx]

**Supplementary Table S1.** The MIC values of reline and parent substances choline chloride and urea against representative strains of Gram-positive and Gram-negative bacteria.

| **Strain** | **Parent substances**  [mmol/mL] | | **Reline**  [mmol/mL] |
| --- | --- | --- | --- |
|  | **Choline chloride** | **Urea** |  |
| *Pseudomonas aeruginosa* PAO1 | 1 | 2.5 | 0.6 |
| *Pseudomonas fluorescens* DSM 50090 | 2 | 1.25 | 0.6 |
| *Klebsiella pneumoniae* Z4 | 2 | 1.25 | 0.6 |
| *Escherichia coli* P4-7250 | 2 | 2.5 | 0.6 |
| *Acinetobacter baumannii* CRAB | 1 | 1.25 | 0.3 |
| *Acinetobacter baumannii* RUH134 | 1 | 1.25 | 0.6 |
| *Staphylococcus aureus* Z7 | 2 | 1.25 | 0.6 |
| *Enetrococcus faecalis* Z9 | 2 | 2.5 | 0.6 |
| *Bacillus subtilis* ATCC6633 | 2 | 2.5 | 1.2 |


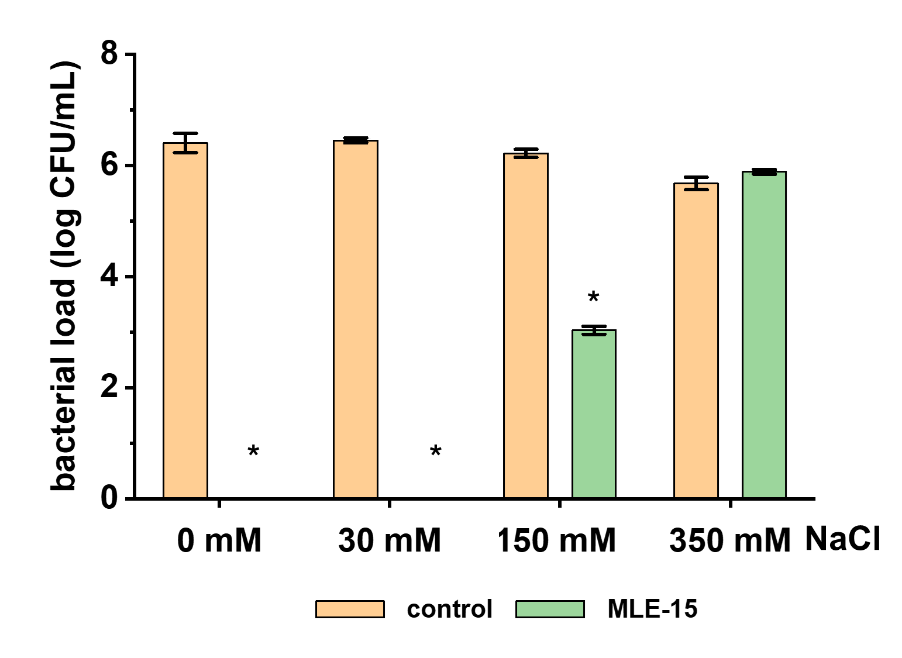


**Supplementary Figure S1.** Killing assay with *Acinetobacter baumannii* RUH134 in buffers with varying tonicities. 20 mM HEPES pH 7.4 was used in this experiment with no salt, or supplemented with 30 mM (hypotonic buffer), 150 mM (isotonic buffer), and 350 mM NaCl (hypertonic buffer). Assay was performed according to Vázquez et al., 2024. The absence of error bars indicates that all three replicates had cell counts below the detection limit, indicating maximum killing activity. Asterisks indicate that cell counts were significantly lower than the control (*p* < 0.05).


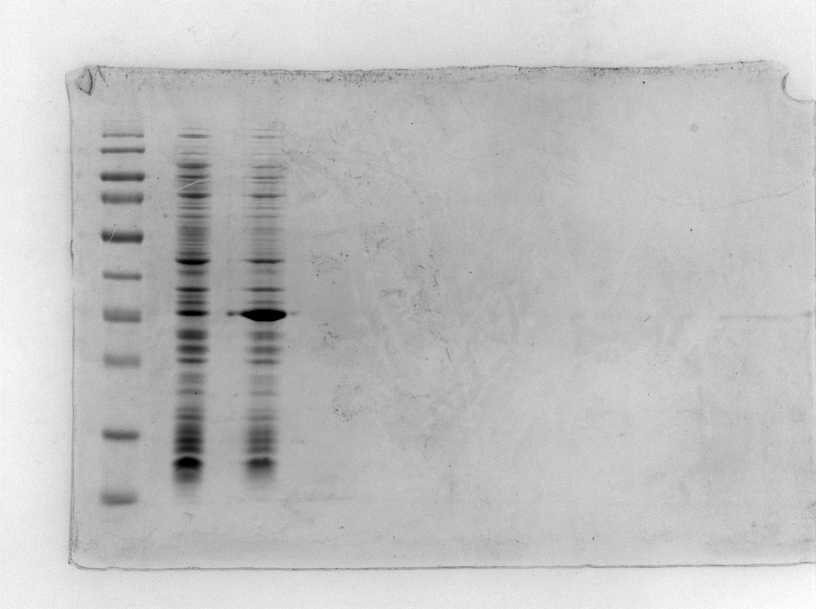


A


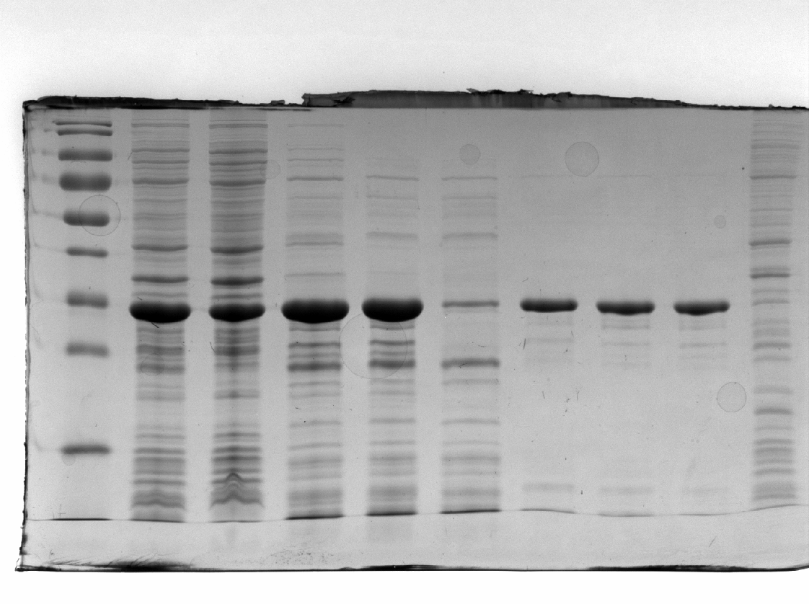


B

**Supplementary Figure S2.** Uncropped images of gels related to Figure 5. Overproduction (A) and purification (B) of MLE-15. See also the RepOD repository (https://doi.org/10.18150/OWFIIK).
